# Theoretical basis, state and challenges of living cell-based drug delivery systems

**DOI:** 10.7150/thno.99257

**Published:** 2024-08-19

**Authors:** Wei Liu, Guowang Cheng, Hao Cui, Zhen Tian, Bowen Li, Yanhua Han, Jia-Xin Wu, Jie Sun, Yuyue Zhao, Tongkai Chen, Guangtao Yu

**Affiliations:** 1 Stomatological Hospital, School of Stomatology, Southern Medical University, Guangzhou, 510280, China.; 2 Science and Technology Innovation Center, Guangzhou University of Chinese Medicine, Guangzhou 510405, China.

**Keywords:** Living cells, Targeted drug delivery, Drug loading approaches, Controlled drug release, Clinical transformation

## Abstract

The therapeutic efficacy of drugs is determined, to a certain extent, by the efficiency of drug delivery. The low efficiency of drug delivery systems (DDSs) is frequently associated with serious toxic side effects and can even prove fatal in certain cases. With the rapid development of technology, drug delivery has evolved from using traditional frameworks to using nano DDSs (NDDSs), endogenous biomaterials DDSs (EBDDSs), and living cell DDSs (LCDDSs). LCDDSs are receiving widespread attention from researchers at present owing to the unique advantages of living cells in targeted drug delivery, including their excellent biocompatibility properties, low immunogenicity, unique biological properties and functions, and role in the treatment of diseases. However, the theoretical basis and techniques involved in the application of LCDDSs have not been extensively summarized to date. Therefore, this review comprehensively summarizes the properties and applications of living cells, elaborates the various drug loading approaches and controlled drug release, and discusses the results of clinical trials. The review also discusses the current shortcomings and prospects for the future development of LCDDSs, which will serve as highly valuable insights for the development and clinical transformation of LCDDSs in the future.

## Introduction

The bioavailability of traditional drugs is severely limited by their short half-lives, poor ability to penetrate physiological barriers, and lack of targeting ability, which result in their low therapeutic efficacy and severe toxicities following administration at higher doses 1. Extensive efforts have therefore been made over the past few decades for developing drug delivery systems (DDSs) that can overcome these challenges 2. Nano DDSs (NDDSs) represent one of the most superior DDS, and have produced significant breakthroughs in clinical practice. Notably, certain commercially available nanomedicines have been utilized for the effective delivery of conventional drugs and specific regulatory elements 2, 3.

In particular, it is important to emphasize the pivotal role of messenger RNA nanomedicines in addressing the challenges posed by the coronavirus disease 2019 pandemic 4. In addition, NDDSs can be functionally modified to ensure the controlled release of drugs during the targeted treatment of lesions. For instance, in order to induce apoptosis in cancer cells using CO and stimulate anti-tumor immune responses, researchers have designed a hollow, rough-surfaced nanoplatform that can carry CO-prodrugs and responsively release CO in the tumor microenvironment (TME) 5. However, conventional NDDSs may exhibit certain challenges during drug delivery, including immunogenicity, biodegradability, formation of nanoparticle-protein corona complexes, side effects caused by the products of decomposition, presence of tissue residues, and first-pass effect in the liver. Altogether, these reveal that the efficiency of NDDSs necessitates further improvement 6, 7.

To address the limitations of NDDSs, researchers have employed the principle of biomimicry and developed advanced drug delivery technologies by encapsulating drugs within endogenous biomaterials that primarily include cell membranes and extracellular vehicles (EVs). The biomaterials used for drug delivery inherit certain physiological functions from parent cells and provide unique advantages for drug delivery, including immune escape potential, prolonged circulation, and specific chemotaxis. Endogenous biomaterial DDSs (EBDDSs) are highly editable and have received extensive attention for drug delivery 8. EBDDSs are prepared by combining different types of cell membranes, including the membranes of living cells and tumor cells. It enables the drugs to acquire the natural characteristics of cancer cells and the specific functions of the proteins from other cell types, which ensures effective tumor targeting and improves the therapeutic effects of drugs 9. However, EBDDSs have some serious limitations, including the disruption of cellular integrity by the biomimetic procedure. Additionally, the application of mechanical forces such as ultrasound or extrusion for drug encapsulation deforms the shape of the reassembled membrane coating and causes irreversible damage to the membrane proteins of cell membrane-based DDSs for encapsulating drugs 10. EBDDSs also affects the efficiency of targeted delivery, which primarily manifests as a marked reduction in the yield of endogenous biomaterials 11 and the absence of organelles that generate the requisite energy for chemotactic movement 12. Altogether, these findings indicate that despite the promising prospects of application, there are several major challenges to the clinical translation of EBDDSs in drug delivery.

Owing to their sensitivity to the pathological environment and simpler processes of extraction, LCDDSs have a more efficient targeting potential than EBDSSs 13. LCDDSs exhibit excellent biocompatibility properties and can actively sense the complex microenvironment *in vivo*, which enables the precise targeting of focal tissues. For instance, macrophages can deliver drugs into lesions and subsequently polarize into the anti-inflammatory or pro-inflammatory phenotype to assist therapy 14. Nowadays, more and more cells types have been used to construct LCDDS, depend on the particularity of their physiological functions, including red blood cells (RBCs), platelets, macrophages, neutrophils, and stem cells 15. The review summarizes the characteristics of NDDSs, EBDDSs, and LCDDSs to better demonstrate the advantages of LCDDSs (Table 1). In particular, the unique advantage of LCDDSs lies in the inherent therapeutic effect of the living cells from which they are prepared, which can be further enhanced by combining LCDDSs with drugs to achieve a synergistic therapeutic effect 16. For example, macrophages load attenuated* Salmonella* for tumor target therapy, which can activate the tumor immune response following administration. This effectively suppresses the biological characteristics of *Salmonella,* while employing the tumor targeting and antigen presenting functions of macrophages 17. Therefore, LCDDSs have attracted extensive attention and have been applied for treating a variety of diseases. The applications of LCDDSs, as reported in relevant literatures 13, 18, 19. Despite extensive studies, there is still a lack of a comprehensive summary of the technology, especially for the detailed description of the drug loading and release process.

**Figure 1:**
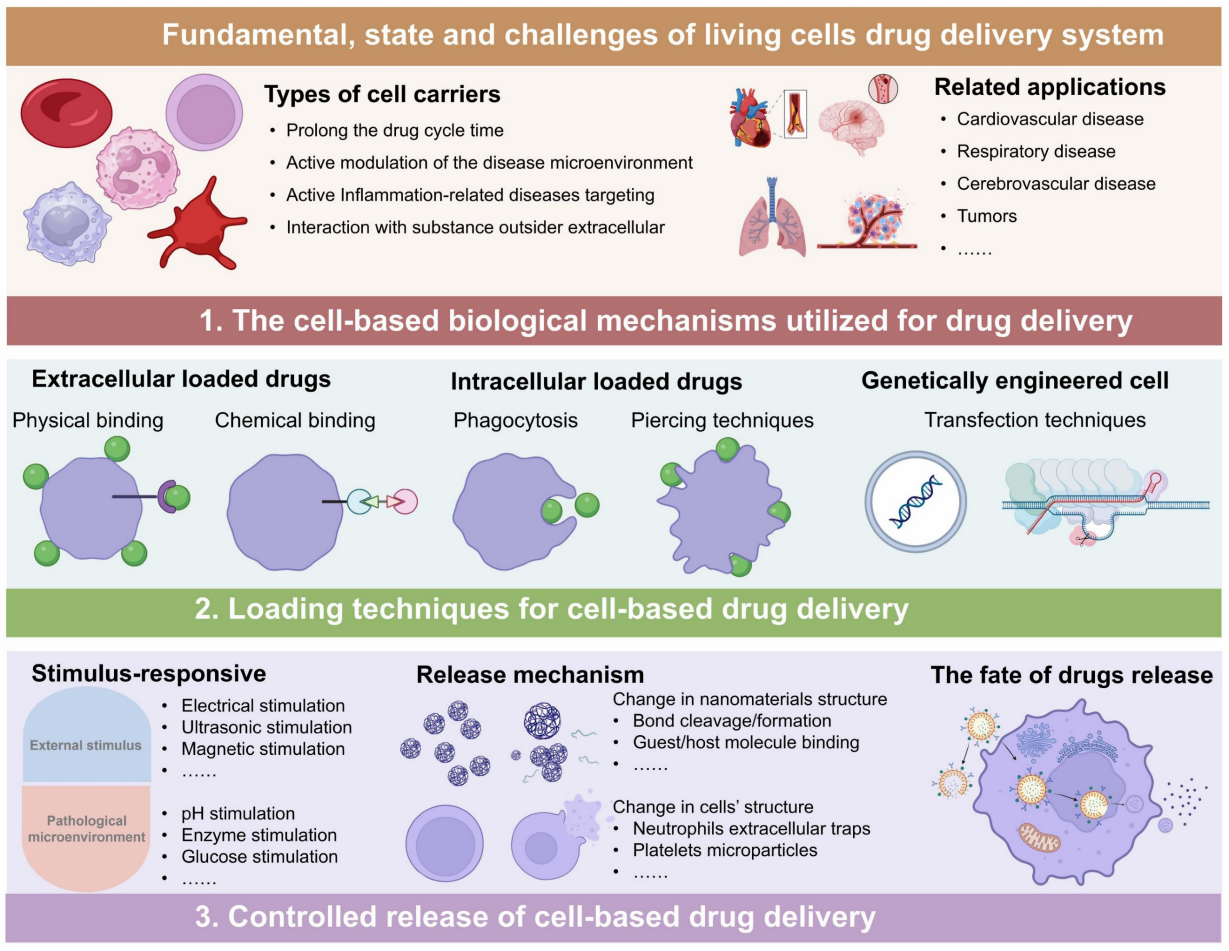
Living cell drug delivery system, from application to loading techniques to controlled release (Figure was created with BioRender.com).

In this review, we systematically discuss the complete processes of LCDDSs, including the principles and mechanisms involved in the design, release, and application of LCDDSs, and reviews the present status, challenges, and future prospects of LCDDSs. As described in Figure 1, the review initially summarizes the mechanism of application of LCDDSs in various disease models based on the physiological functions of living cells. Furthermore, the review lay emphasis on describing the design strategies involved in drug loading and release, followed by a detailed summary of the clinical trials on the development of LCDDSs. Finally, we discuss the problems and challenges and present our views and recommendations. This review aims to promote the technological development and clinical translation of LCDDS to ensure the safe and efficient delivery of drugs to the target site.

## The biological functions of LCDDS

**Figure 2:**
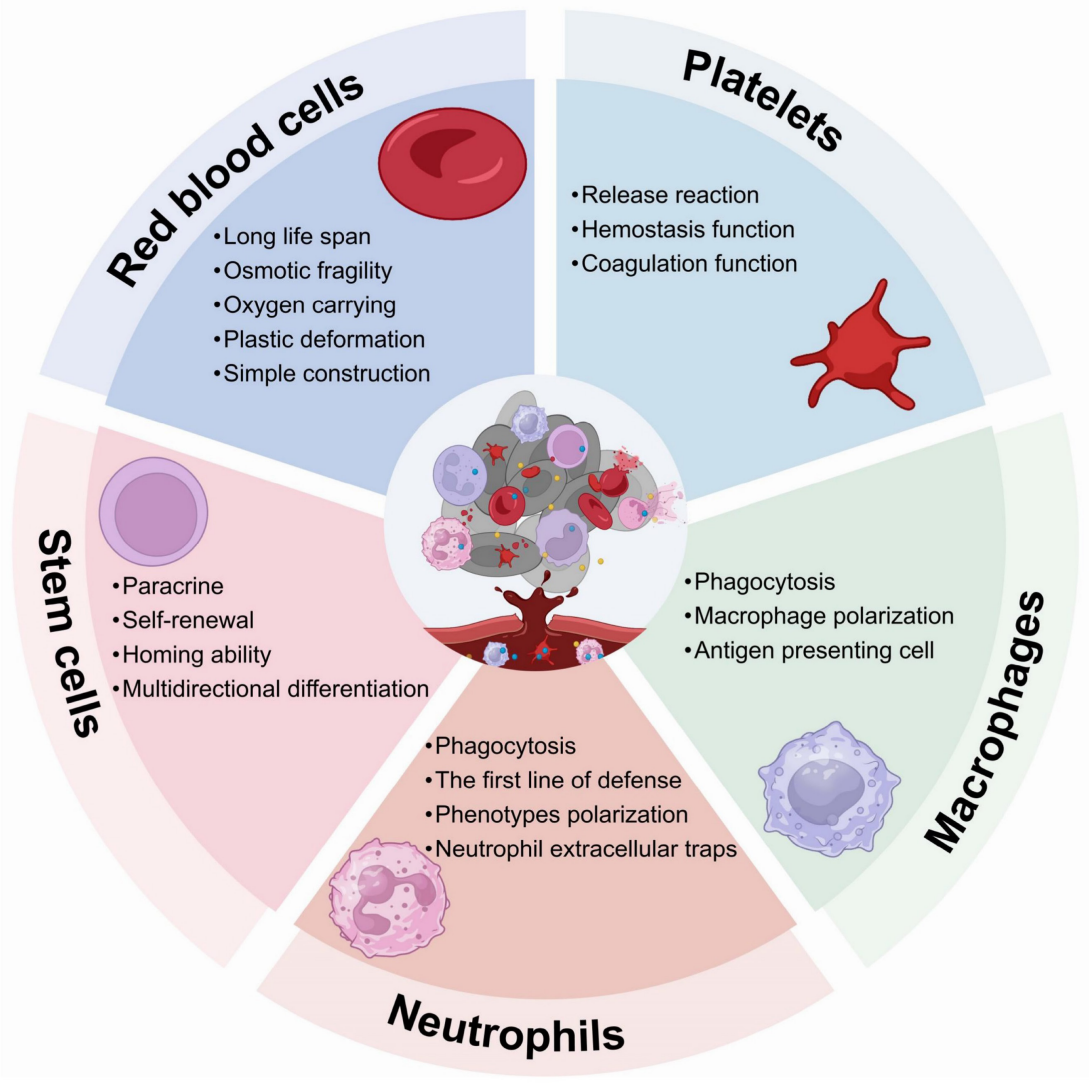
The properties of various living cell carriers (Figure was created with BioRender.com).

Living cells, such as RBCs, platelets, neutrophils, macrophages and stem cells, play an important role in maintaining immunological homeostasis. Moreover, they actively participate in disease treatment. Consequently, these cells have been investigated as functional carriers for loading and delivering drugs to various diseases. With continuous advancements, LCDDS are gradually towards maturity. The distinctive biological characteristics of living cells serve as crucial determinants that directly influence their behavior and physiological functions *in vivo*. The carrier cells commonly used and their properties, functions are showed in Figure 2. Moreover, this section provides a detailed discussion from the cellular biological functions to the application of LCDDS in disease therapy. We also summarize the biological characteristics and application mechanisms of different cell carriers (Table 2), while Table 3 outlines the delivery techniques and current applications of living cells.

### Red blood cells

RBCs, the most abundant cells in the blood, are derived from hematopoietic stem cells (HSCs) in the bone marrow and enter the bloodstream after maturing. RBC-based DDS make full use of the properties of RBCs, especially, their long-term stability within the systemic circulation. It make them an ideal materials for biomedical applications 20.

Firstly, mature RBCs have a biconcave shape and a high surface-to-volume ratio, while lacking nuclei and organelles. The exceptional elasticity and plasticity of RBCs enable them to maintain cellular integrity under mechanical pressure when traversing capillaries 21. These distinctive characteristics provide ample capacity for loading inner cargoes and surface modification 22, facilitating the hitchhiking of drugs by RBCs and their release under varying osmotic pressures. It has been demonstrated that conjugates of small molecule anticancer drugs with polyzwitterions can reversibly bind to the RBC membrane, which facilitates extravasation into the tumor interstitium via the enhanced permeability and retention effect 23. Another advantage of using RBCs is that they have a lifespan of 120 days in the circulation 24. Additionally, the expression of the cluster of differentiation (CD) 47 glycoprotein on the RBC membrane transmits an inhibitory “don't eat me” signal via the signal regulatory proteins alpha (SIRPα) pathway, which enables their prolonged *in vivo* circulation without clearance by mononuclear phagocytic cells 25. The prolonged circulatory ability of RBCs provides a novel therapeutic strategy for the administration of short half-life drugs that require frequent injections, which enables the stable release of drugs* in vivo*, while addressing the issues related to unstable blood concentration and side effects caused by frequent injections.

Based on the aforementioned physiological characteristics of RBCs, they sever as highly desirable carriers for drug delivery. The primary function of circulating RBCs are transporting oxygen from the alveoli to the tissues by hemoglobin and eliminating carbon dioxide after gas exchange 26. This unique ability allows RBCs to efficiently reach all organs and tissues, providing a biological basis for targeting drugs delivery to downstream organ via bloodstream 27 and treatment of vascular embolism disease 28. Although further investigation is required to fully comprehend the mechanism behind organ-specific targeting, it can be demonstrated that RBCs-based DDS have been applied to systemic therapies with good prospects.

In addition, RBCs are also involved in immunological functions. The surface receptors of RBCs (*e.g.,* complement receptor, toll-like receptors 9 (TLR9), CD71^+^ erythroid cells) have been found to interact with various inflammatory molecules and involve in immune responses. The immune mechanism of RBCs involves the combination of antigen-antibody complexes with immune components present on RBCs' surface. These complexes are transported to the liver and spleen, where they promote macrophages phagocytosis, leading to the uptake and clearance of immunological components. Eventually, RBCs either dissociate from the complex and return to the bloodstream 29, or undergo phagocytosis by macrophages if their morphology changes or lack of CD47 30.

The RBC-based DDS fully utilize the known physiological functions and properties of RBCs. However, limited attention has been dedicated to RBCs' senescence and oxygen carrying capacity, which necessitates further exploration. There is a decrease in the expression of membrane surface proteins and enzymes associated with senescent RBCs, resulting in reduced flexibility and increased fragility. These alterations render them easily recognizable and phagocytosed by macrophages, primarily accumulating in the spleen and liver, followed by the bone marrow and other body tissues 31. Taking inspiration from the characteristics of senescent RBCs, we can design them to load drugs for targeted accumulation in the liver, spleen and bone marrow for treating corresponding organ disease. Additionally, the occurrence of hypoxia in the disease environment, especially within tumors, poses as a significant challenge to treatment. Hypoxia is known to promote disease progression and significantly alter cellular viability. RBCs serve as exclusive carriers and transporters of oxygen. In recent years, research efforts have predominantly focused on modifying hemoglobin to create artificial RBCs and enhance the oxygen-deprived conditions at disease sites 32, 33. Furthermore, this discovery has inspired researchers to explore the potential of utilizing natural RBCs for improving oxygen delivery in hypoxia environments. However, RBCs are susceptible to fatigue as the treatment cycle prolongs, which leads to hemolysis. The phenomenon can be mainly attributed to the deformation and recovery during each compression, which is companied with the release and recovery of adenosine triphosphate (ATP). When ATP release exceeds ATP recovery, ATP depletion will cause the dissociation of the band 3-anchor protein bonds between the cell membrane and cytoskeleton. Consequently, morphological change occur in RBCs along with a gradual loss of deformability, ultimately happen hemolysis 34. Another issue of using RBCs is that the excessive embellishment of drugs on the RBC surface can reduce their elasticity and deformability, and this poses as a serious disadvantage when it is accompanied by the loss of the extracellular CD47 molecules. This may potentially trigger undesirable clearance of RBCs by and even lead to RBCs agglutination.

### Platelets

Platelets are anucleate membrane cell fragments derived from megakaryocytes and exhibit a significantly smaller size compared to other nucleated blood cells 35. They express and secrete molecular regulators involved in thrombosis and inflammation, thus playing a crucial role as mediators of hemostasis and involve immune responses 36. Platelets contain three types of granules. The first type, known as alpha granules (α-granules), predominantly contain proteins, many of which serve as primary immune regulatory molecules, including platelet factor 4 and transforming growth factor-β. The second type, referred to as dense granules, mainly comprise small molecules including calcium, ATP and epinephrine that are primarily related to thrombosis. Lastly, the third type consists of lysosomal granules that play an important role in initiating tissue remodeling and protein degradation 36, 37. When sense an inflammatory environment, the signals interact with receptors on platelets surface, and subsequently activated platelets. The activated platelets undergo a morphological transformation from their discord shape to a spherical conformation accompanied by the formation of lamellipodia, which facilitates their interaction with other cells. And the release of platelet-microparticles (PMPs), including shedding fragments, dense granules and α-granules, thereby further amplifies platelets activation. The relocation of glycoproteins such as CD40 and P-selectin from cytoplasmic granules to the surface of activated platelets contributes to the interaction with immune cell 38, 39. Therefore, the function of platelets largely depends on their release response, which mediates their roles in hemostasis, thrombosis, inflammation, tissue regeneration promotion, and tumor progression.

It has long been thought that platelets primarily contributes to maintain the primary hemostasis and blood flow within vessel 37. In their resting state, circulating platelets exhibit a discoid shape and do not interact with the intact vessel wall 40. However, upon endothelial dysfunction, they can rapidly undergo activation, leading to a change in morphology and increased adhesiveness to vascular walls within minutes. Activated platelets aggregate and form thrombus through interaction with fibrin in order to seal ruptured vessels 41. Thus, platelets-based DDS for loading thrombolytic drugs has emerged as an effective therapeutic strategy for treating thrombotic diseases 42.

In addition to their crucial roles in coagulation and the maintenance of hemostasis following mechanical injury to the vasculature, platelets contain a plethora of bioactive molecules in their granules and express different receptors on their surface that contribute to the progression of cancer and metastasis 43. Angiogenesis is a crucial phenomenon driving tumor cell metastatic dissemination 44. One of the characteristics of tumor-associated vascular systems is the formation of immature and permeable vessels, which leads to local hemorrhaging, activation of the coagulation cascade, and the recruitment of platelets 45. Recent studies have demonstrated that the inhibition of immune checkpoints is beneficial for patients with malignancies. Platelet-based DDSs have therefore been specifically designed to enable the efficient delivery of immune checkpoint inhibitors for the treatment of tumors. Tumor cells express the programmed death-ligand 1 (PD-L1) protein that interacts with programmed death-1 (PD-1) receptor on immune cells, primarily T cells, which leads to the exhaustion of immune cells and helps tumor cells evade surveillance, and consequent immunosuppression 38. Platelets loaded with anti-programmed cell death-ligand 1 (aPD-L1) particles for their subsequent delivery to the tumor site, which triggered the release of PMPs containing immune aPD-L1. The findings revealed that the administration of PD-L1-loaded platelets effectively attracted other immune cells and thus validated the successful application of the platelet-based DDS in a murine model for achieving targeted drug delivery to metastatic tumors and enhancing the efficacy of the loaded drug 45. In addition, platelets can actively target circulating tumor cells (CTCs) to facilitate the formation of PLT-tumor cell aggregates via the interaction of P-selectin and CD44 46. The resulting aggregates exert pro-metastatic or anti-tumor growth effects depending on the specific characteristics of the TME 47. Therefore, the unique interaction between platelets-CTCs can be harnessed to develop novel strategies for targeting CTCs and preventing tumor metastasis. This approach has demonstrated the delivery of apoptosis inducers (such as membrane attack complex 48 and tumor necrosis factor-related apoptosis-inducing ligand 49) to mimic immune cell-mediated tumor killing, as well as antiplatelet drugs 50 to inhibit platelets and consequently prevent platelets from supporting CTCs, thereby delaying tumor metastasis.

Platelets have also been interacted with immune regulation 51. During inflammatory conditions, activated platelets directly interact with circulating leukocytes through P-selectin and P-selectin glycoprotein ligand-1, CD40 and CD154, thereby facilitating the formation of platelet-leukocyte aggregates and promoting leukocytes rolling at sites of inflamed or damaged endothelium. The interaction contributes significantly to both innate and adaptive immunity 36, 52. Notably, platelets contain a multitude of pathogen-associated molecular pattern recognition receptors, such as toll-like receptors, nucleotide oligomerization domain-like receptors and c-type lectin receptors. These receptors serve as pivotal regulators in the identification of pathogen and initiation of innate immune responses 53. Thus, platelets can be harnessed to devise drug delivery carriers for treatment of immune-mediated inflammatory diseases 38, 51, exert anti-inflammatory effects 54 and facilitate wound healing.

Platelets as drugs carriers, not only concern with their physiological functions, but also require careful consideration of activation and release responses. However, platelets tend to aggregate at the site of vascular injury and bind coagulation factors and tissue factor to achieve hemostasis, which can lead to thrombosis formation. Failure to administer timely thrombolytic will pose the risk of vascular embolism, when loading drugs onto platelets for the treatment of vascular diseases, thrombolytic therapy should be taken into account. Activated platelets are also implicated in tumor growth, angiogenesis, and immune evasion, in addition to facilitating the adhesion and colonization of circulating tumor cells on the distal endothelium 55. Relevant studies have also demonstrated that platelets can shield tumor cells from immune surveillance 56. Therefore, the application of platelets as drug carriers may inadvertently promote tumor development. These findings highlight that the exploration of alternative strategies is imperative for mitigating the risks associated with the application of platelets as DDSs.

### Neutrophils

Neutrophils originate from HSCs and developed in the bone marrow, which are the most abundant type of leukocytes in the vascular system. The growth, synthesis, and release of neutrophils into circulation are primarily regulated by granulocyte colony-stimulating factor (G-CSF). Mature neutrophils are retained in the bone marrow through interaction with C-X-C chemokine receptor (CXCR) 4, while their release from the bone marrow is facilitated by CXCR2. The balance between retention and release is regulated by G-CSF 57. Neutrophils are widely recognized as the primary line of defense against pathogens in the innate immune system 58, 59, exhibiting rapid responsiveness to inflammation through activation, adhesion, and migration from endothelial vessels to inflammatory tissues 60.

Neutrophils detect and engulf pathogens by recognizing pathogen-associated molecular patterns (PAMPs) 14. Once sensing inflammatory stimuli and chemoattractant, activated neutrophils will migrate the focal tissues and amplify their recruitment and/or activation through C-X-C chemokines or leukotriene B4 and integrin adhesion. This ability enables neutrophils to deliver anti-inflammation drugs to inflammatory sites. Previous studies have used neutrophils for delivering of paclitaxel for postoperative glioma and cerebral ischemia 61, 62, drugs delivery for atherosclerosis 63, among others. Neutrophils contribute to the development of pathologic venous and arterial thrombosis by releasing neutrophil extracellular traps (NETs) 64, and the inhibition of NETs may reduce the formation of pathological thrombosis; however, the inhibition of neutrophils remains controversial owing to their critical role in the immune response. Therefore, studies aimed at harnessing the potential targeting ability of neutrophils for delivering thrombolytic drugs in thrombotic diseases have received increasing attention 15. Senescent neutrophils exhibit a pro-inflammatory phenotype that is characterized by various cellular changes, including the upregulation of CXCR4, which facilitates the retention of neutrophils in the bone marrow and delays their entry into the circulation 57. This phenomenon enables the targeted delivery of drugs to pathological sites within the bone marrow using neutrophils 65.

In addition, numerous researches have demonstrated the significant role of neutrophils in tumor occurrence, development and treatment. Neutrophils can either promote or inhibit tumor growth depending on their polarization state. Several factors within the TME influence the polarization of neutrophils toward distinct phenotypes. Based on their polarization status, two populations of tumor-associated neutrophils (TANs) have been identified: N1 neutrophils with a short lifespan and mature phenotype, exhibiting anti-tumor ability through increased production of tumor necrosis factor (TNF), intercellular adhesion molecule, reactive oxygen species (ROS), CD95, as well as downregulation of arginase expression. On the contrary, N2 neutrophils have a limited lifespan, immature phenotype, promoting tumor growth through the secretion of arginase and hydrogen peroxide 66. Considering the dual role of neutrophils in tumors, utilizing neutrophil-based drug delivery can effectively treat tumors by polarizing their phenotype and combining with therapeutic drugs. However, when employing neutrophils for mediating drug delivery to tumors, it is crucial to fully consider the phenotype of N1 neutrophils. This is because using untreated neutrophils as carriers may pose a risk where drug-carrying neutrophils could be reprogrammed into the immunosuppressive pro-tumor N2 phenotype within the TME after homing to tumor sites 67.

All of these neutrophils-based DDS have shown excellent targeting capabilities and therapeutic efficacy. Nonetheless, the short lifespan of neutrophils and limited technological advancements pose challenges. Despite attempts to alter the phenotype of neutrophils, in practice neutrophils designed *in vitro* may undergo unknown phenotype reprogramming when injected into the complex disease microenvironment.

### Macrophages

Macrophages play crucial roles in recognizing, phagocytosing, and eliminating invading pathogens to initiate innate immune responses. As professional antigen-presenting cells (APCs), they possess the capability to internalize, process, and present antigens to facilitate adaptive immune reactions 68.

These inherent physiological functions promote macrophages to be used as drug delivery vehicles.

In this process, macrophages are initially recruited to inflammatory tissues through chemokines and cytokines guidance. They recognize and engulf foreign materials via TLR or other pattern-recognition receptors that allow them to spontaneously prepare drugs 69. The ability of macrophages to access inflammatory tissues and cross the blood-brain barrier, make them treat inflammation-relation disease, such as Parkinson's disease 70, pneumonia 71, live injury 72 and other pathological sites. Subsequently, macrophages degrade foreign products and express on the surface of macrophages in the form of peptides-major histocompatibility (MHC) II complex, which are present to CD4+ T cell. Macrophages also present peptides- MHC I complex to CD8+ T cell by antigen cross-presentation pathway 73. Therefore, the interaction between macrophage and T cells is considered a critical communication between the innate and adaptive immune systems 74. Emphasizing the importance of macrophages in delivering drugs to selectively eliminate infected cells and facilitate T cell activation, thereby initiating an adaptive immune response. For example, macrophages can be loaded with chemotherapeutics that have been demonstrated to induce immunogenic cell death (ICD). The ICD induction, acting as “*in situ* vaccines”, is captured by macrophages and dendritic cells, subsequently presented to T cells, thereby effectively stimulating an immunogenic hot TME 75. The approach also elicits a robust immune response effectively suppress tumor metastasis and recurrence 76. This innovative approach presents a novel strategy for designing macrophages as carriers capable of inducing ICD for achieving effective tumor immunotherapy. Although similar methods are relatively few studies devoted to anti-inflammation and anti-viral, this way holds promising potential for future applications.

It is noteworthy that SIRPα, an inhibitory receptor, is prominently expressed on the surface of macrophages. CD47 is widely expressed on various cells, particularly tumor cells and pathological cell 77. The interaction between CD47 and SIRPα effectively prevents macrophages phagocytosis. Consequently, targeting the CD47/SIRPα axis has emerged as a promising therapeutic strategy for suppressing tumors 77, treating cardiovascular disease 78, steatohepatitis 79 and other pathological condition.

To be more precise, macrophages exhibit remarkable plasticity and can differentiate into either M1 or M2 macrophages, thereby producing distinct types of cytokines. M1 macrophages are involved in pro-inflammatory and anti-tumor properties, while M2 macrophages play a role in anti-inflammatory responses and tumor promotion. This phenomenon of the two distinct phenotypes of M1/M2 polarization is commonly referred to as “macrophage polarization” 80. M1 macrophages are typically induced by Th1 cytokines, such as lipopolysaccharide and interferon-γ stimulation, which result in cellular flattening and secretion of pro-inflammatory cytokines including interleukin-1α (IL-1α), IL-1β, tumor necrosis factor-α, *etc*. On the other hand, M2 macrophages are polarized by Th2 cytokines like IL-4 and IL-13, leading to cell elongation and secretion of anti-inflammatory cytokines such as IL-10, transforming growth factor-β, arginase-1, among others 81, 82. Considering the phenotypic reversal of macrophage at disease sites and their inherent physiological function, inducing phenotypic polarization through macrophages-based drug delivery represents an effective strategy for treating various diseases. It has been observed that macrophages can be actively recruited to tumors through diverse mechanisms in the formation of tumor-associated macrophages (TAMs), predominantly consisting of M2 macrophages that promote tumor progression while creating a suppressive immune microenvironment 83. Thus, macrophages or M1 macrophages have been designed as carriers for loading anti-tumor drugs to specifically target tumors, thereby not only exerting therapeutic effects but also inducing repolarization of endogenous TAMs into anti-tumor M1 macrophages to enhance anti-tumor treatment. Currently, M2 macrophages play a crucial role in inflammatory diseases. They payload effectively reduces inflammation by serving as both an anti-inflammatory drug and repolarize M2 macrophages immunoregulation 14. Reprogramming either M1 or M2 macrophages holds great promise as a potential approach for treating tumors or inflammatory diseases.

Due to their diverse physiological functions, macrophages-based DDS offer multiple pathways to treat various diseases. Furthermore, these functions are not utilized independently within DDS, instead, there exist interconnections and combinations among them that promote the advancement of macrophages-based DDS. However, the drug degradation that caused by macrophage phagocytotic ability need to be improved, as well as ensuring the preservation of phenotypic state in M1 or M2 macrophages following reversal.

### Stem cells

Stem cells possess inherent homing properties, multidirectional differentiation potential, self-renewal capacity, and paracrine effects 84, rendering them highly promising in the realms of tissue engineering, regenerative medicine, and drug delivery. According to the developmental potential stem cells can be divided into totipotent stem cells (TSCs), pluripotent stem cells (PSCs) and multipotent stem cells 85. TSCs refer to single cells capable of forming a complete organism, the fertilized egg cells are the prime example^86^. PSCs, including human embryonic stem cells (hESCs), human induced pluripotent stem cells (hiPSCs), can differentiate into cells of all three germ layers 87. The mesenchymal stem cells (MSCs) are derived from the mesoderm of hESCs 86. The multipotent stem cells can differentiate into closely related cell lineages such as HSCs and neural stem cells (NSCs).

Several studies have demonstrated the regenerative and reparative potential of stem cells in cardiomyocytes 88, pancreatic β-cells 89, hepatocytes 90, *etc*. Thus, offering promising avenues for improving or treating cardiovascular diseases, liver diseases, and diabetes. Additionally, there are widespread concern in inducing hiPSCs or adult MSCs to develop organoids, which can reproduce the cellular heterogeneity, structure and function of human organs 91. Consequently, under specific conditions, stem cells can be induced to differentiate into specialized cells types, making them highly attractive candidates for targeted drug delivery 18.

In addition to their differentiation and tissues repair capabilities, stem cells can also involve in the development of various disease through their homing and paracrine properties. MSCs are widely distributed throughout the human body 92. Besides, MSC exhibit multilineage differentiation potential into osteoblasts, chondrocytes, adipocytes and other lineages 93. Therefore, MSCs are one of the most extensively studied cell types in the field of stem cell DDS. Extensive literatures have reported that MSCs loaded drugs can improve the delivery of antitumor drugs 94, cytokines 95, and therapeutic genes 12 in a variety of tumors models. It is widely accepted that the exogenous MSC targeting to TME is guided by a chemokine gradient. These chemokines include a variety of cytokines and their corresponding receptors, which secreted by tumor cells and other immune cells in the TME, such as vascular endothelial growth factor/vascular endothelial growth factor receptor, stromal cell-derived factor 1/CXCR4 96. Upon adherence to tumor endothelial cells, MSC secretes matrix metalloproteinases 2 (MMP-2) matrix protease to mediate their migration. Subsequently, MSCs exert immunomodulatory functions through direct interaction with immune cells and paracrine effects. They effectively suppress immune cells activation, promote the expansion of regulatory T cells, and affect macrophage polarization, thereby facilitating the establishment of immune tolerance and inhibiting cytokine storm 97, 98. Based on the immune function of MSCs, the utilization of gene delivery vectors to modify MSCs enable engineered MSCs to express immunomodulatory capabilities 12. MSCs have also been used to carry anti-inflammatory drugs 99 to target and treat inflammatory diseases.

HSCs are present in the bone marrow, umbilical cord blood and constitute a vital component of the circulatory system. They possess remarkable self-renewal and replication capabilities, enabling them to differentiate into diverse hematopoietic and glandular cell lineages. Ultimately, they give rise to various blood cell constituents such as RBCs, leukocytes, and Platelets, thereby ensuring long-term homeostasis of both the hematopoietic and immune systems 100. Initially, HSCs transplantation was extensively employed for the treatment of hematological malignancies 101. However, the occurrence of chronic graft versus host disease (cGVHD) post-HSCs transplantation poses a significant obstacle to HSCs development. To overcome the immune barrier associated with hematopoietic stem cell transplantation, a novel therapeutic strategy involves genetic modification of autologous hematopoietic stem cells to serve as a source for other blood cells 100. This approach utilizes HSCs as a carrier for delivering therapeutic genes and inducing their differentiation. While the surface proteins of the modified HSCs remain intact, their genetic material is altered, thereby virtually eliminating rejection. This approach increases the potential for targeting a wide range of diseases. For example, studies have demonstrated that lentivirus-infected HSCs can be expanded and differentiated into enucleated engineered RBCs expressing tumor-specific peptides bound to MHC-I along with co-stimulatory ligands and IL-12 on their surface. These modified activate T-cells and promote antigen-specific T-cell expansion, thus enhancing anti-tumor immunotherapy 102.

NSCs, which are exclusively found in the central nervous system, possess the ability to differentiate into neural tissues such as neurons, astrocytes and glial cells 103. Additionally, NSCs exhibit remarkable properties including amyloid-β and tau protein 104 clearance as well as tumor homing capabilities 105 Therefore, NSCs-based DDS have been explored for treating neurological diseases including glioblastoma 106 and Alzheimer's disease 107.

Overall, stem cell drug delivery therapy effectively utilizes its paracrine, multidirectional differentiation, and homing properties. However, considering the heterogeneity and source of stem cells, safety needs to be considered before clinical use, and precise induction of stem cell differentiation is needed in the future.

Cell drugs conjugates can be designed to release stimulatory factors to enhance cell proliferation, persistence, or activity. And drugs and cytokines can also be combined and loaded cells, which achieve intracellular drug loading and extracellular cytokine modification, thereby enhance the targeting ability 108. Additionally, cells often play dual roles in diseases, and rationally modifying cells and drugs can promote their transformation into therapeutic phenotypes.

## Drug loading techniques of LCDDS

**Figure 3:**
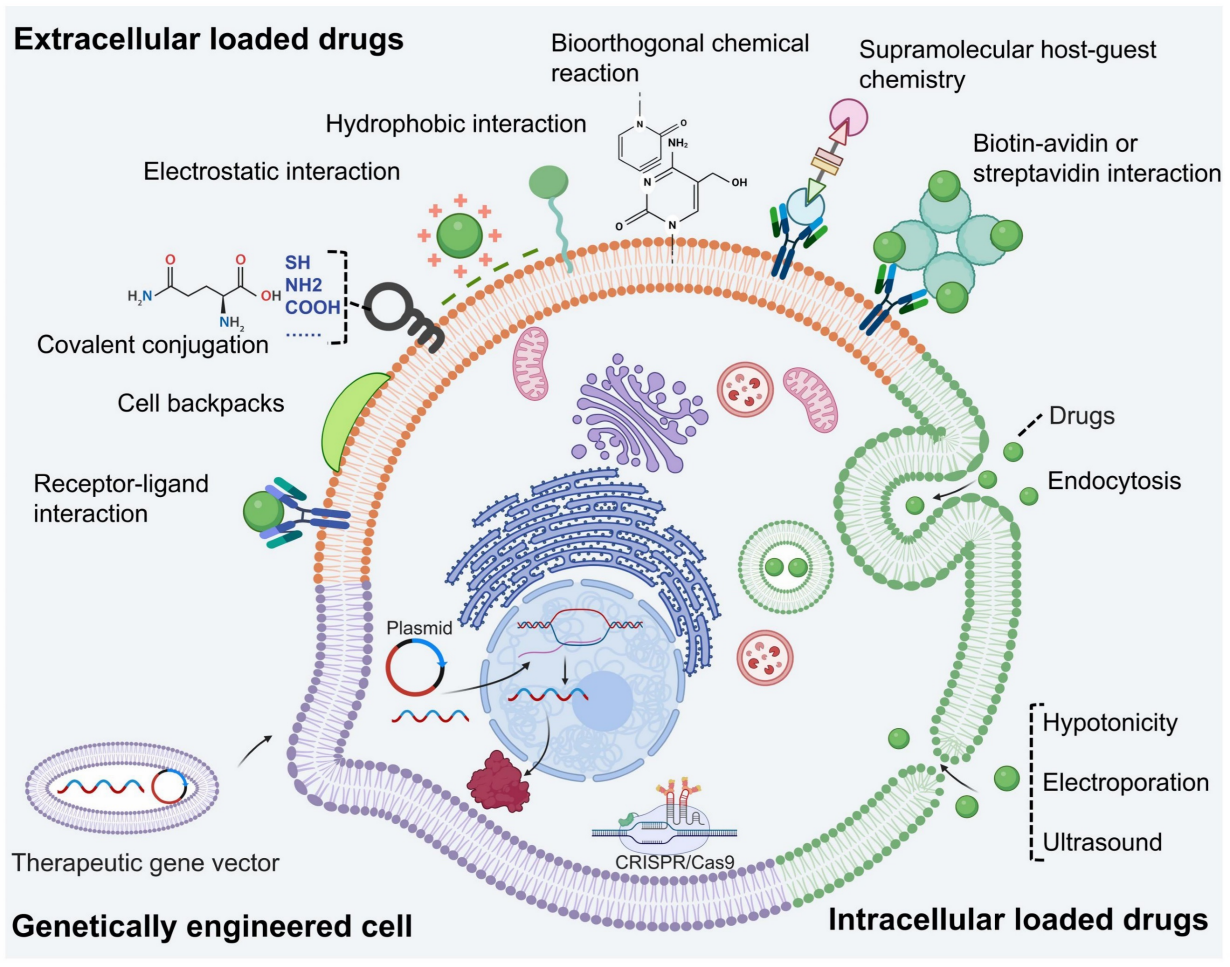
Drug loading strategies-based living cells, encompassing extracellular and intracellular drug loading approaches, as well as genetically engineered (Figure was created with BioRender.com).

LCDDS offers significant advantages in drug loading due to the ample space and functional sites provided by living cells (Figure 3). Considering the properties of cell membranes, drugs can be loaded onto the cell surface through physical or chemical binding methods. Moreover, intracellular drug loading in the cytoplasm can help avoid off-target effects. Additionally, cell engineering techniques enable modification or enhancement of original cellular functions. In the process, it is necessary to ensure precise delivery of drugs to the targeted disease site without affecting the cell activity and minimizing off-target effects. Therefore, this subsection provides a comprehensive elucidation on the principles and methodologies of three loading techniques for LCDDS.

### Extracellular drug loading

Currently, the technology of extracellular drug loading can be categorized into two parts: physical binding and chemical binding, based on whether the surface of living cells is modified. Physical binding does not modify the cell surface but imparts drug new structure to enable it to bind to the cell surface. And chemical binding introduces new biomolecules or groups onto the living cell surface without affecting its biochemistry. These biomolecules or groups allows for selective and specific modifications at desired sites on the cell membrane surface. Besides, drugs loaded onto cell surface protect drug against intracellular enzymes, maintain cellular membrane integrity, and avoid interference with internal biological process.

#### Physical binding

The cell membrane is a biological interface consisting of phospholipid bilayer, saccharides and proteins. Its surface carrier a large number of ligands and receptors and groups 141. The cell membrane is negatively charged on the outside, while the interior is hydrophobic 142. Therefore, drug formulations can be loaded on the cell surface through various methods such as receptor-ligand interactions, covalent coupling, electrostatic interactions, and hydrophobic interactions.

**Figure 4:**
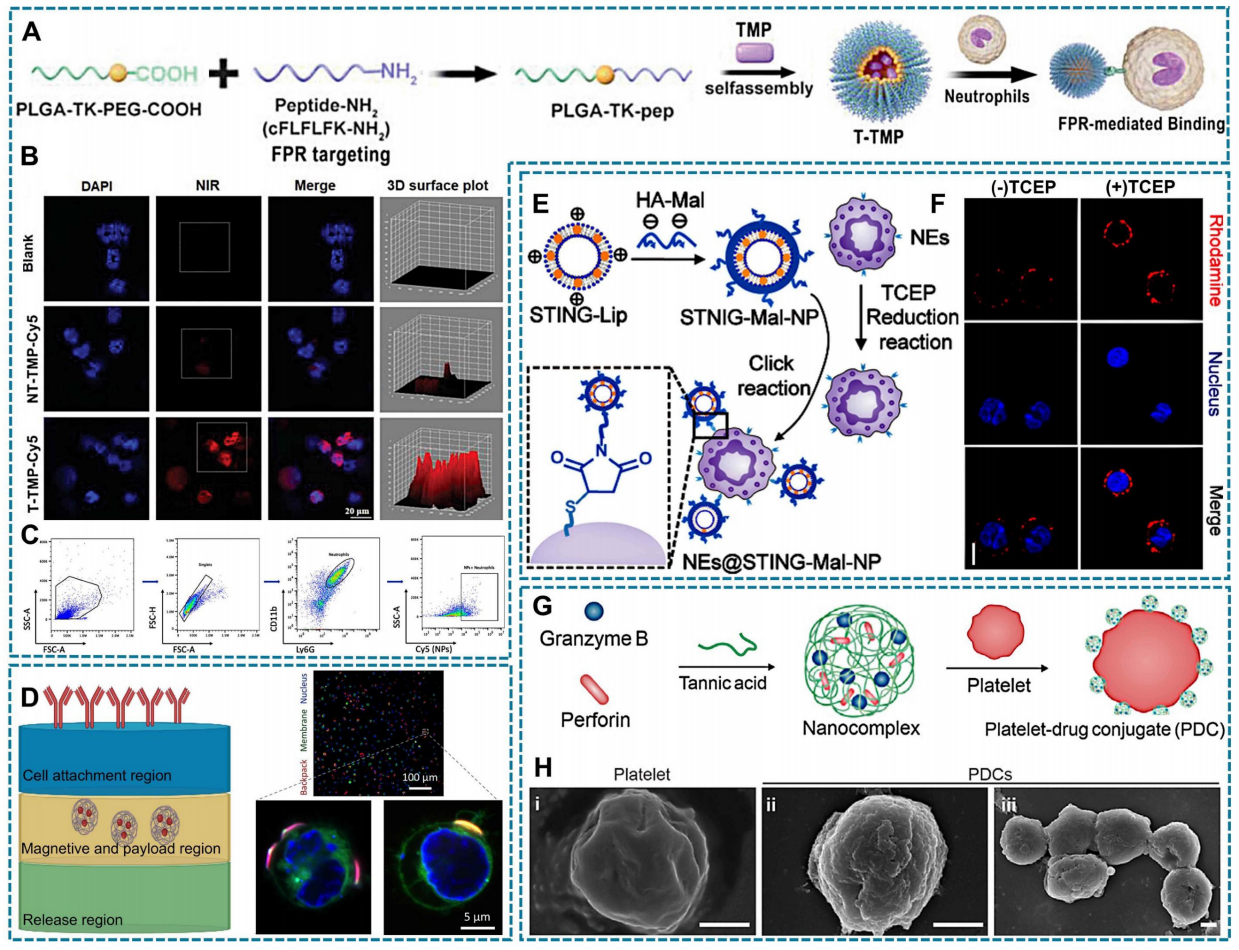
Relevant illustration of drug loading techniques involving physical binding with living cells.** (A)** T-TMP was synthesized through self-assembling TMP and FPR targeting peptide with PLGA-TK-PEG-pep, followed by specific targeting neutrophils through the FRP receptor. **(B)** Confocal images exhibit neutrophils have a enhanced red fluorescence of indocyanine green Cy5-labeled T-TMP and NT-TMP. **(C)** Flowcytometry results shows the uptake of T-TMP by neutrophils. (A-C Reproduced with permission 60 Copyright 2023, Wiley-VCH GmbH). **(D)** (left)Three layers cellular backpack assembly; and (right) Confocal micrographs of cells (nucleus, blue; membrane, green; backpacks: red). (Left, created with BioRender.com; Right, Reproduced with permission 145 copyright 2020, The American Association for the Advancement of Science).** (E)** The scheme of NEs@STING-Mal-NP were synthesized by the covalent binding between Mal on the surface of STING-Mal-NP and the thiols on the surface of neutrophils after reduction reaction by TCEP. **(F)** Confocal microscopy image of neutrophils with or without TCEP reduction after incubation with STING-Mal-NP-Rhodamine (red). Nucleus was stained by Hoechst (blue) (E-F Reproduced with permission 151 copyright 2023, American Chemical Society). **(G)** The tannic acid encapsulates granzyme B and perforin formed nanocomplex, and the electrostatic interaction or hydrophobic interaction between the polyphenol moieties of nanocomplexes and platelet surface.** (H)** The SEM images of platelets and PDCs, which demonstrated that PDCs had a rougher surface structure compared with unmodified platelets. (G-H Reproduced with permission 48 copyright 2023, Elsevier).

The specific binding and interaction between receptors and ligands form the foundation of cellular communication with the external environment, such as immune responses and signal transduction. Receptor-ligand interactions occur through non-covalent binding without requiring chemical modification, relying primarily on the specificity and affinity between the receptor and ligand. Consequently, it becomes feasible to modify drug surfaces with ligands that selectively bind to cell membrane receptors 143, 144. For instance, formyl peptide receptor (FPR) present on the surface of neutrophils. The therapeutic drug tetramethylpyrazine (TMP) is encapsulated in a novel polymer via ROS-sensitive thioketal (TK) bond that connects poly (lactic-co-glycolic acid) (PLGA) and polyethylene glycol (PEG). The carboxyl terminal of PEG is linked to the peptide cinnamyl-F-(D) L-F-(D) L-F (CFLFLF), enabling specific targeting of neutrophil FPR (Figure 4A). Compared to NT-TMP (TMP without CELFLF) and blank groups, neutrophils in the T-TMP (TMP with CELFLF) showed a higher uptake of T-TMP by neutrophils as confirmed through confocal microscopy and flow cytometric analysis (Figure 4B-C) 60.

Cellular backpacks (BPs) are a class of soft discoidal particles that can bind on the surface of cells 145. Previously, BPs were primarily designed with three layers, which were constructed using layer-by-layer (LBL) assembly-method 146, and load with drugs bound to cell surface via various cell surface conjugation approaches 147. Folate receptor (FR) can be utilized as a mediator for attaching cellular backpacks to macrophage surfaces 148. As shown in Figure 4D, the “release region” is capable of dissolution when desired. The "magnetism and payload region" can accommodate drugs. And the "cell attachment region" contains antibodies enabling specific attachment of backpacks to desired cell types 148, 149. Currently, the strategy of BPs has been optimized on the basis of three layers. The two layers of BPs can directly bind to cells by LBL assembly formed an adhesive nanocoating 137.

Covalent conjugation capitalizes on the presence of naturally occurring reactive groups, such as amine and thiol groups, on the cell surface, facilitating the formation of a covalent bond with a complementary reaction partner on the NP surface 150. As an example, hyaluronic acid-maleimide (HA-Mal) can form covalent bonds with thiols, and Zhang *et al*., developed Mal-coated liposomes with stimulator of interferon genes (STING) agonists. Simultaneously, tris (2-carboxyethyl) phosphine (TCEP) was employed to expose free thiols on the surface of neutrophils, enabling the covalent binding of Mal-coated liposomes to neutrophils surfaces (Figure 4E). Besides, Rhodamine-labeled Mal-coated liposomes exhibited enhanced fluorescence intensity in neutrophils with TCEP reduction thiols (Figure 4F) 151.

Electrostatic interaction is based on the electrostatic adsorption between cationic materials and electronegative cell surface. Positively charged nanomedicines may bind to negatively charged glycosaminoglycans on the cell membrane, or they may bind to negatively charged lipids on the cell membrane 116. Due to the hydrophobic nature of the cell membrane surface, upon contact with hydrophobic elements, they undergo aggregation and adsorption. Among them, aromatic amino acids can form π: π with unsaturated fatty acids and adsorb on the cell surface 152, 153. Furthermore, similar to the principle of solubility, drugs containing lipid components create favorable conditions for their integration into cell membranes 154. The nanocomplexes loaded with tannic acid encapsulating granzyme B and perforin, as shown in Figure 4G, demonstrate the formation of platelet-drug conjugates (PDCs). These nanocomplexes establish electrostatic or hydrophobic interactions between their polyphenol moieties and the surfaces of platelets. Compared with natural platelets, engineered PDCs had a rougher surface, which demonstrated successful drug loading (Figure 4H) 48.

Although these methods are relatively mild and simple, the off-target effect of LCDDS may lead to unexpected side and toxic effects 155. The physical and chemical properties of drugs, such as hydrophobicity and charge distribution, determine their interaction with the components of the cell surface. When the protein corona adheres to the surface of drugs, it may impact pharmaceutical activity and even shield the crucial responsive targets, hindering drug release. Additionally, the protein corona can also alter drug function, such as shortening half-life and reducing the interaction with targets 156.

#### Chemical binding

Chemical binding artificially introduces groups or specifically modifies cell surface to achieve selectively loading drugs. Currently, available chemical binding includes supramolecular host-guest chemistry, bioorthogonal chemical and biotin-avidin or streptavidin interaction.

**Figure 5:**
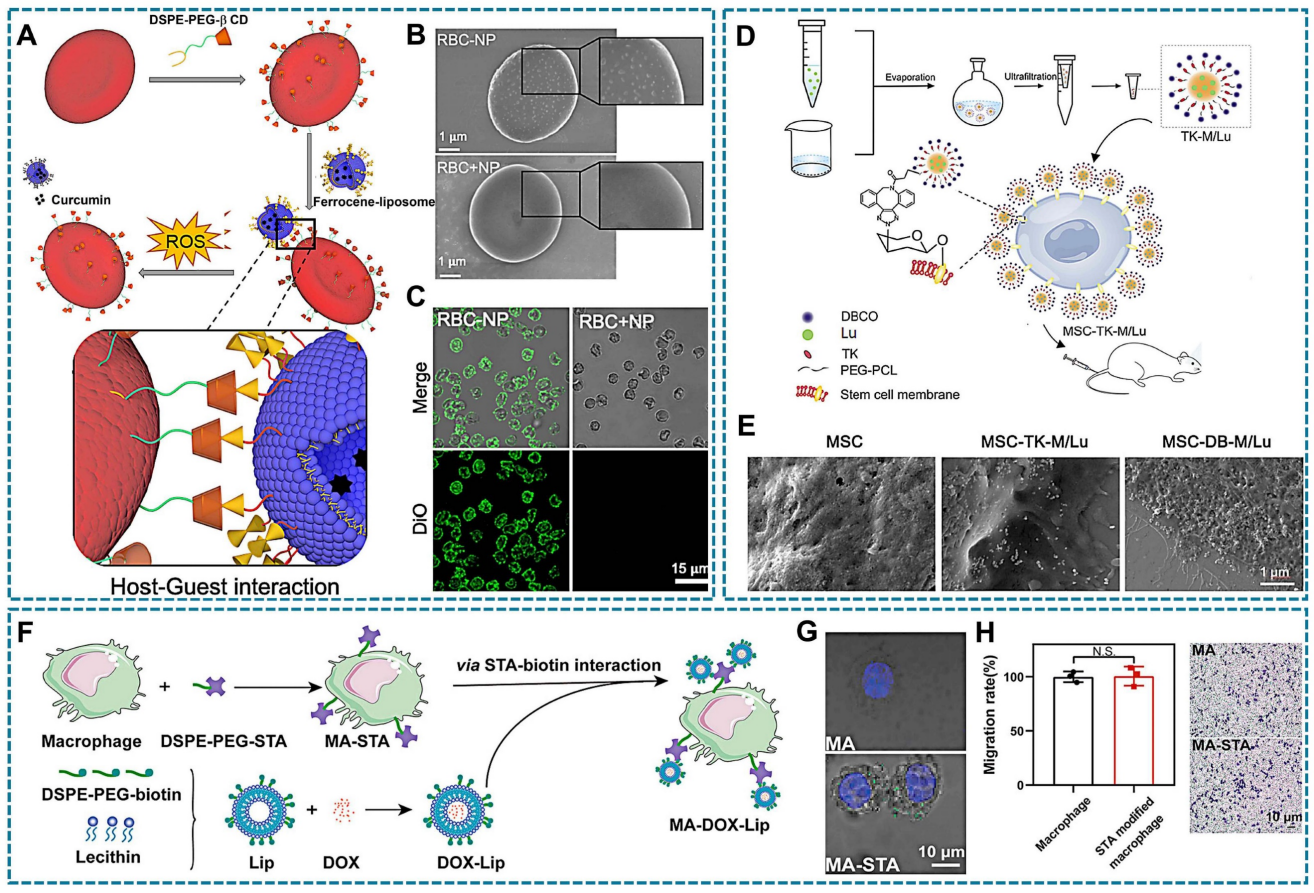
Relevant illustration of drug loading techniques involving chemical binding with living cells. **(A)** Schematic representation of the host-guest complex formed by β-CD and ferrocene. β-CD, β-cyclodextrin.** (B)** SEM and **(C)** CLSM images are obtained for red blood cells (RBCs) incubated sequentially with DSPE-PEG-CD and ferrocene-NP (RBC-NP), as well as RBCs coated with Fc-NP (without β-CD) on their surface (RBC+NP). The green fluorescence indicated the presence of DIO-loaded ferrocene-NP. (A-C Reproduced with permission 160 copyright 2022, Elsevier). **(D)** Schematic illustration of click chemistry reaction. TK-M/Lu were prepared by using solvent evaporation method and subsequently tethered onto the surface of MSCs via bioorthogonal click chemistry.** (E)** SEM images revealed the attachment of luteolin-loaded micelles with (MSC-DB-M/Lu) or without DBCO (MSC-TK-M/Lu), to the surface of MSCs. Respectively. (D-E Reproduced with permission 99 copyright 2023, Elsevier). **(F)** Schematic representation of the fabrication procedure for macrophage-liposome complexes. The DSPE-PEG-biotin-modified liposomes are conjugated to streptavidin (STA)-modified macrophages through STA-biotin interaction. **(G)** CLSM images of the physical mixture of FITC-PEG-biotin and DSPE-PEG-STA-modified macrophages, while the undecorated cells showed no significant attachment.** (H)** Evaluation of the migratory capacity of macrophages with and without DSPE-PEG-STA modification revealed that surface modification did not significantly impact macrophage motility, as evidenced by a cell migration assay (F-H Reproduced with permission 132 copyright 2022, American Chemical Society).

Supramolecular host-guest chemistry has been explored in DDS, involving host macromolecules, guest units and host-guest complex 157. Commonly employed host macromolecules such as cyclodextrins, calixarenes, cucurbiturils, and pillararenes possess a cone-shaped structure with a hydrophilic outer surface and a hydrophobic inner cavity 158. Moreover, certain guest macromolecules like ferrocene, adamantane, and its derivatives can be encapsulated within the cavities of host macromolecules to form supramolecular polymers through intermolecular non-covalent interactions 159. Due to the strong binding affinity between the host and guest components, this supramolecular complex exhibits excellent stability in LCDDS. In this process, 1,2-Distearoyl-sn-glycero-3-phosphorylethanola mine (DSPE) and PEG are usually used as link vehicles for modifying host macromolecules and guest macromolecules 147. As shown in Figure 5A, β-cyclodextrin (β-CD) with DSPE -PEG modification was inserted into the RBCs membrane, while β-CD bind ferrocene (Fc) liposomes nanoparticle (NP) with loading curcumin. Moreover, the Fc-NP could be observed attachment in RBCs surface (Figure 5B-C) 160.

Bioorthogonal chemical reactions involve the labeling of bioorthogonal groups on cell membranes under physiological conditions, enabling selective binding of cargo with complementary groups through bioorthogonal reactions. These reactions can be rapidly and selectively conducted in a biological environment without interfering with normal physiological processes of living cells. Due to their simplicity and specificity, bioorthogonal chemistry holds significant potential for applications in cell labeling, target recognition, drug delivery, *etc*. Metabolic precursors such as amino acids, monosaccharides, and choline are essential for the synthesis of proteins, glycans, and phospholipids on cell membranes. To facilitate their incorporation into cellular metabolic pathways and subsequent presentation on the cell membrane, these precursors can be modified with bioorthogonal groups like N_3_, dibenzyl cyclooctyne (DBCO), trans-cyclooctene, and tetrazine. Consequently, therapeutic agents equipped with complementary groups can be selectively targeted to the cell surface through bioorthogonal reactions 161. Click chemistry is an indispensable member of bioorthogonal chemical reaction. It achieves intermolecular linkages through ring-forming reactions involving carbon-heteroatom-carbon bonds. The widely used copper-catalyzed azide-alkyne cycloaddition (CuAAC) has been limited by the cytotoxicity of copper catalysts, leading to the development of catalyst-free click chemistry alternatives such as strain-promoted azide-alkyne cycloaddition and inverse electron demand Diels-Alder reaction 162. These advancements provide novel strategies for targeted in LCDDS. An example is that anti-inflammation drugs encapsulated by micelles with DBCO-PEG-TK-PCL polymer, preparing luteolin-loaded micelles (TK-M/Lu). And MSCs were pre-incubated with Ac_4_GalNAz to obtain MSCs expressing the azide group (N_3_-MSCs). Subsequently, the micelles could be bioorthogonal conjugated onto the surface of metabolically glycoengineered MSCs was observed by scanning electron microscope (SEM) (Figure 5D-E) 99. In addition, dendritic cells as the key cells that mediate adaptive immune responses. It was labeled chemical tags by metabolic glycoengineering, and effectively capturing the antigen/adjuvant with DBCO modification, then achieve targeted vaccine design for dendritic cells 163. This method can also be explored for macrophages that another type of APCs.

The non-covalent coupling of biotin-avidin/streptavidin interaction is widely recognized as the most robust binding between proteins and ligands 164, making it highly applicable in various fields such as drug delivery, immunoassays, and polymerase chain reactions due to its exceptional stability and selectivity. Biotin, a water-soluble vitamin essential for natural growth and development of the body, plays a crucial role in these interactions. Avidin are glycoproteins present in hen egg white, while streptavidin is a protein secreted by Streptomyces that shares structural and functional similarities with avidin. Both proteins consist of four subunits, each capable of binding one molecule of biotin with exceptionally high affinity 165. The tetrameric structure of avidin or streptavidin remains intact even under extreme pH conditions, denaturing agents, and enzymatic degradation 166. Biotin also contributes to the structural stability of avidin/streptavidin through its interaction with these proteins. Moreover, the formation of the avidin/streptavidin-biotin complex occurs rapidly in aqueous solutions at room temperature, making it suitable for applications involving cells sensitive to organic solvents and temperature 150. Biotin exhibits simultaneous binding to all four binding sites of avidin or streptavidin, thereby minimizing the percentage of binding mismatches. Moreover, the utilization of avidin or streptavidin as a bridging agent facilitates the assembly of multiple biotinylated components and significantly broadens the applications. And can be employed to target the cellular surfaces. For instance, Yang *et al*., designed and synthesized DSPE-PEG-streptavidin and biotin liposomes, wherein DSPE-PEG-streptavidin was incorporated into the macrophage membrane to expose streptavidin, formed macrophage-streptavidin (MA-STA). Additionally, doxorubicin, a chemotherapy drug, was encapsulated within biotin-presenting liposomes that were attached to macrophages through biotin-streptavidin interaction (Figure 5F) 132. Under confocal microscopy, it can be observed that fluorescently labeled biotin rapidly attaches to the cell surface, while unmodified cells have no obvious attachment. In addition, the surface modification had no effect on the motility of macrophages (Figure 5G-H). Moreover, the interaction between biotin and avidin/streptavidin facilitates robust immobilization of drug loading on the cell surface, effectively addressing the issue of unstable loading that can impact cellular signaling pathways. However, this strong immobilization complex poses challenges in terms of dissociating and releasing drugs at the lesion site. Additionally, it is important to consider the choice between avidin and streptavidin due to avidin's non-specific binding caused by its strong positive charge and glycosyl chain. Conversely, streptavidin overcomes these non-specific binding limitations.

The process of chemical binding modifies the structure of both cells and drugs, enhancing the weak binding strength and reducing drugs off-target effects. However, excessive modification of cells carriers and drugs may produce additional binding sites, leading to non-specifically interactions. Furthermore, it disrupts intercellular interactions, leading to the clearance of living cells. Therefore, it is necessary to find a reasonable and moderate surface modification strategy to minimize the off-target effect, accurately target diseases, and reduce potential side effects while ensuring pharmaceutical activity.

### Intracellular drug loading

The cytoplasm offers ample internal space for drugs loading and prevents drugs from detaching from the cell membrane, which is benefit for decreasing off-target effect. To achieve this goal, various internalized pathway such as phagocytosis and endocytosis, and some physical strategies can be exploited. Therefore, a comprehensive understanding of these internalization pathways is crucial for intracellular drug delivery. This subsection presents detailed information on intracellular drug loading.

#### Internalization process

Cells can allow nutrients to cross the cell membrane and enter the cytosol through different internalization pathways, including phagocytosis, micropinocytosis, and endocytosis.

**Figure 6:**
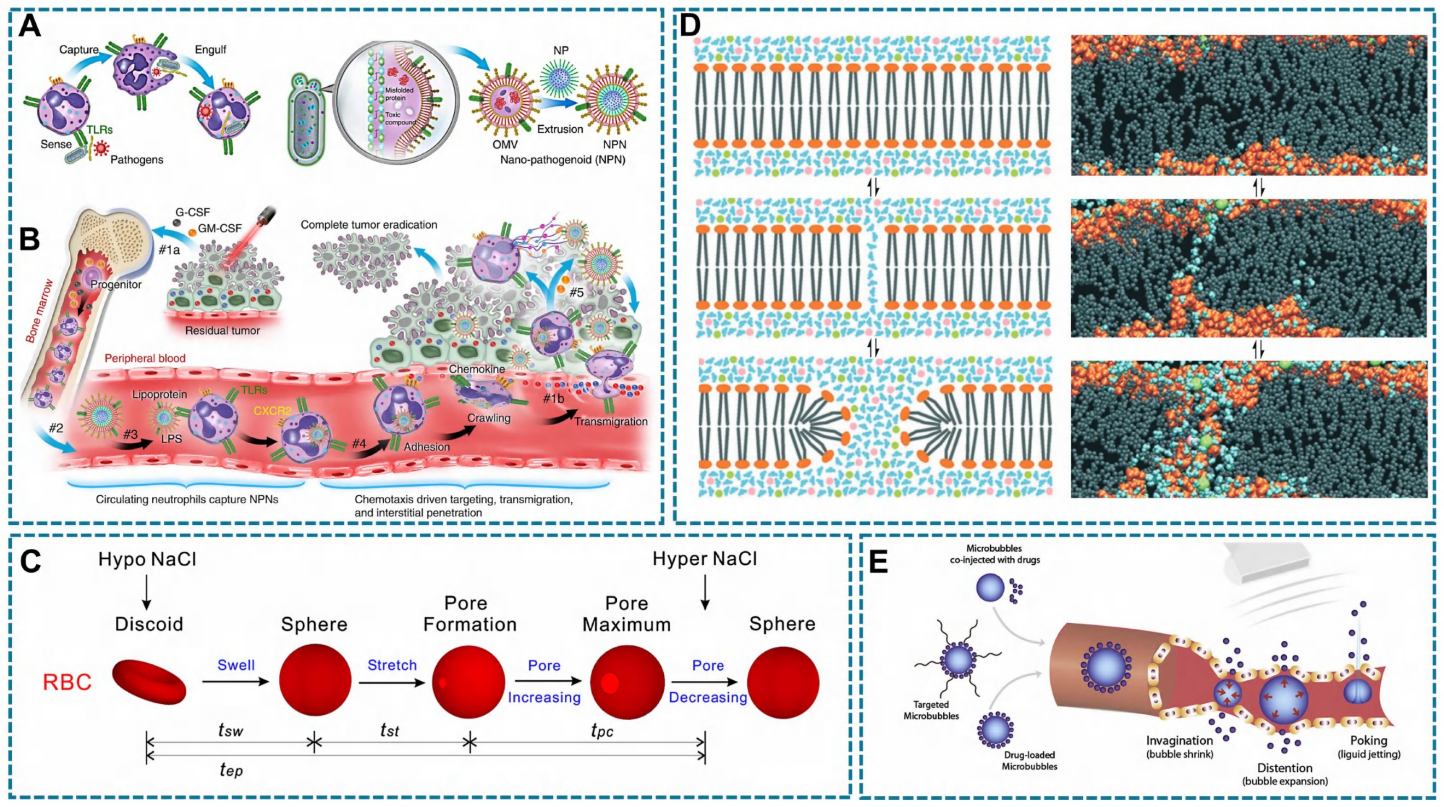
Relevant illustration of drug intracellular loading techniques with living cells.** (A)** Neutrophils sense, capture, and engulf pathogens by recognizing the PAMPs with TLRs (left), Preparation of NPNs by coating OMVs on NPs, which inherit PAMPs from the OMVs (right).** (B)** Treatment-induced cell death created an inflammatory environment of the residual tumor and induced the production of G-CSF, GM-CSF, and chemokines CXCL1 and MIP-2. #1a The released G-CSF and GM-CSF increased neutrophil production from bone marrow. #1b The released CXCL1 and MIP-2 broadcasted the location of the inflamed tumor. #2 Neutrophils entered the blood circulation and encountered the injected NPNs. #3 Neutrophils sensed NPNs with the recognition of LPS and lipoprotein by TLRs and subsequently engulfed them. #4 Neutrophils laden with NPNs were recruited into the tumor site in response to the chemokine gradient through the following cascade: adhesion, crawling and transmigration. #5 NPNs were released from neutrophils to kill tumor cells along with the formation of NETs in the inflamed tumor (A-B Reproduced with permission 130 copyright 2020, Springer Nature). **(C)** Schematic of the erythrocyte shape change in the drug-loading process: tsw, tst, tpc, and tep denote the swelling time, stretching time, pore opening-closing time, and loading time, respectively (Reproduced with permission 173 copyright 2018, Springer Nature).** (D)** Rearrangement of the cell membrane at the molecular level (left) and atomic level (right) under an electric field. Intact bilayer. Intact bilayer (top). Process of water molecules penetrating the bilayer (middle). Reorientation of lipids (bottom) (Reproduced with permission 178 copyright 2022, Royal Society of Chemistry). **(E)** Schematic diagram of ultrasound-guided drug delivery. Ultrasound triggers microbubble oscillation (expansion and shrinkage) and collapse, causing vessel deformation, rupture and permeability change, which allows efficient drug delivery in spatiotemporally controlled way. (Reproduced with permission 183 copyright 2020, Elsevier).

Phagocytosis is the process by which phagocytes (*e.g*., neutrophils and macrophages) uptake cellular debris, including dead cells, and pathogenic microorganisms 167. During this process, phagocytes extend their membrane (pseudopods) to enclose the pathogen particles, then encapsulate the particles within a pseudopodia vesicle and transport them into the cytoplasm 168. The size of particles that can be internalized by phagocytosis is controversial, it is generally believed that phagocytosis mainly works with particles larger than 500 nm 167. Besides, phagocytes can specifically recognize and hijack bacterial derived particles. Inspired by this, a bacterial outer membrane vesicle (OMVs) coating nano-pathogens (NPNs) were developed. The fabricated NPNs are easy to be recognized and internalized by neutrophils after intravenously injection, and homed to the inflammatory lesion (Figure 6A-B) 130.

Receptor-mediated endocytosis is a complicated pathway that sometimes be mistaken regarded as clathrin-mediated endocytosis (CME), indeed it is composed of several endocytosis pathways, including CME, fast endophilin-mediated endocytosis (FEME), and clathrin-independent/dynamin-independent endocytosis (CLIC/ GEEC) 167. In the CME pathway, particles bind to receptors in the cell membrane and initiate the accumulation of intracellular phosphatidylinositol 4,5-bisphosphate, recruits articulin and actin, leading to the formation of a clathrin-coated pit 169. Compared to the CME pathway, FEME is another rapid endocytosis pathway triggered by specific transmembrane receptors that not dependent on lattice proteins but is dependent on actin 167. CLIC/GEEC endocytosis also occurs through specific ligand-receptor interactions, while it is not dependent on dynamin and lattice proteins and mediates cargo uptake differently than using the FEME pathway. Based on above principle, some LCDDS have been developed for the treatment of various disease in recent years. For example, Yang *et al.,* used mannose-modified polymersomes load methotrexate for rheumatoid arthritis therapy, in which mannose can specially recognize CD206 on the surface of macrophages, result in the initiation of the receptors-mediated endocytosis process and finally construct a macrophage-based DDS 81.

Currently, the shortcomings of internalization-based LCDDS can be divided into two aspects. Firstly, the transport vesicles, the compartment formed after endocytosis, may transport drugs to lysosomes, and lead to the inactivation of drugs. Therefore, lysosomal escape capacity is beneficial to improving the efficacy of LCDDS. Secondly, phagocytes internalize particles through actin mediated membrane movement 170. It is thought that the drugs will not be internalized by cells after electrostatic and hydrophobic interaction. The potential mechanism might be that the parameters of lipid materials are different. For example, negatively charged hydrophobic NPs at low concentrations may do not cause cell membrane structure disturbance 171, hindering the endocytosis processes by inhibiting actin. Additionally, micropinocytosis, another actin-based internalization process, can drive cell membrane folds to extend outward to form irregular endocytosis vesicles 169, may also be present in the construction process of some LCDDS, but detailed studies are yet to be carried out.

#### Hypotonicity

RBCs have osmotic properties, can be contracted or swelled in a hypo/ hypertonic medium 172. Inspired by this, hypotonicity is used to loading drugs into RBCs, and hypotonic pre-expansion and hypotonicity dialysis are common strategies to achieve this goal.

During hypotonic pre-expansion, RBCs go through three phases (Figure 6C): swelling, stretching, and pore appearance and disappearance. In the hypotonic solution, extracellular water flows into the intracellular through the osmotic pressure difference, so that RBCs will be spherical. Meanwhile, the cell membrane area constantly extends, thus the membrane tension reaches a threshold and forms a hemolytic pore to facilitate drug entry into RBCs. Finally, a hypertonic solution was added for a period time to close the pore 173. To maintain cell viability, the fabricated RBCs-based DDS were usually collected by centrifugation 114. The advantages of this strategy are better encapsulation efficiency and preserving the bioactivity and immunological properties of the RBCs membrane 174. The principle of the hypotonic dialysis in RBC-based DDS preparation is similar to the hypotonic pre-expansion, with the difference is the collection process of RBCs-based DDS, in which the RBCs are separated from the solution regulating the osmolality by a semi-permeable membrane.

Hypotonicity does not require modification of the RBCs or the drugs, thereby is widely used in the preparation of RBCs-based DDS. However, this simple preparation process is still disturbed by some factors that impact the final properties and encapsulation rate of carrier RBCs. Firstly, osmotic pores within RBCs can be categorized into reversible and irreversible types. To ensure the integrity of RBCs and minimize hemolysis rates, only reversible pores are considered. Additionally, there is limited literature available on pore diameter which relates to the conversion time point between hypertonic and hypotonic solutions. Then, regarding the diameter of the drugs, especially to nanomedicines, and the loading threshold in the RBCs. RBCs encapsulation process needs to be completed under the operation of three solutions with different osmotic pressure, exploration the optional solution osmotic pressure is necessary. Finally, an ideal RBCs-based DDS needs to ensure that the encapsulated drug does not leak before the pore is closed, and also that keeping the viability of RBCs.

#### Electroporation and Ultrasound

To help the drugs enter the carrier cells, some physical strategies were used to enhance the permeability of the cell membrane. Electroporation is a common method to transiently interferes with cell membrane stability by external electric field 175. The cell can be considered as a simplified circuit model that consists of extracellular resistance (*R_e_*), intracellular resistance (*R_i_*), and the capacitance of the cellular membrane (*C_m_*) 176. In the external electric field, *C_m_* can be regarded as a dielectric capacitor, and potential difference between the *R_e_* and* R_i_* is the transmembrane potential *V_m_.* When the external electric field reaches a certain level, the *V_m_* exceeds the critical transmembrane threshold, then the lipid bilayer appears rearrangement and finally form temporary pores (Figure 6D), so that drugs can be transported into the cell 177. In general, there are three types of electroporation, including conventional bulk electroporation (BEP), microscale electroporation (MEP) and nanoscale electroporation (NEP) 178. Among them, BEP is based on a high frequency current while can directly pass the cell and generate large amounts of heat that cause cell death 179. In contrast to BEP, MEP and NEP improve the uniformity of the electric field distribution with lower frequency current, thus cell damage is avoided while pores were formed 176, 178.

Another strategy to form transient pores is sonoporation triggered by microbubbles under ultrasound treatment 180, in which the microbubbles refer to a lipid, protein or polymer shell stabilized gas-filled structures 181. Under ultrasound condition, microbubbles occur cavitation because of their special gas-filled structures. Furthermore, higher ultrasound intensity induces a stable cavitation process, and the lower ultrasound intensity cause the inertial cavitation 182. Blood vessels undergo deformation, rupture, and changes in permeability under the influence of ultrasound (Figure 6E) 183. Similarly, microvesicles experience morphological alterations induced by cavitation, leading to mechanical compression of the cell membrane and resulting in reversible modifications that facilitate drug penetration into the cell. Besides, a part of the formed pore is attributed to the production of ROS in this process, which is also leading to membrane disruption via lipid peroxidation, and stimulating endocytosis 184.

One challenge of the application electroporation and ultrasound in LCDDS is maintaining the cell viability. Therefore, for forming moderate size and reversible pores in the cell membrane, it should to pay attention to optimize parameters of the external electric field and ultrasound.

### Genetically engineered

Nowadays, cellular products such as cytokines and Evs have emerged as potent therapeutic agents. To enhance the production of these therapeutics, genetically engineered technology is employed to establish therapeutic cells 185. To achieve this goal, viral and non-viral vectors are common tools to edit the targeting cells. The viral vectors can bind to the receptors of host cells, then transported to cytoplasm for releasing loaded genetic materials 186. Although viral vectors provide the higher transmission efficiency, the concern is the non-ideal safety 187. In contrast, non-viral vectors with a better biocompatibility, there are consisted of cationic lipids or polymers, calcium phosphate co-precipitation. The cationic lipids or polymers shell ensures they can be endocytosed and complete the transfection process. The calcium phosphate co-precipitation is prepared by putting DNA and calcium chloride mixture into phosphate solution. The host cells internalize such carrier through the phagocytosis pathway, so that DNA materials can be released in the cytoplasm 186, 187. In addition, single cells directly enhance the permeability of cell membrane and delivery gene sequences into host cells 188.

**Figure 7:**
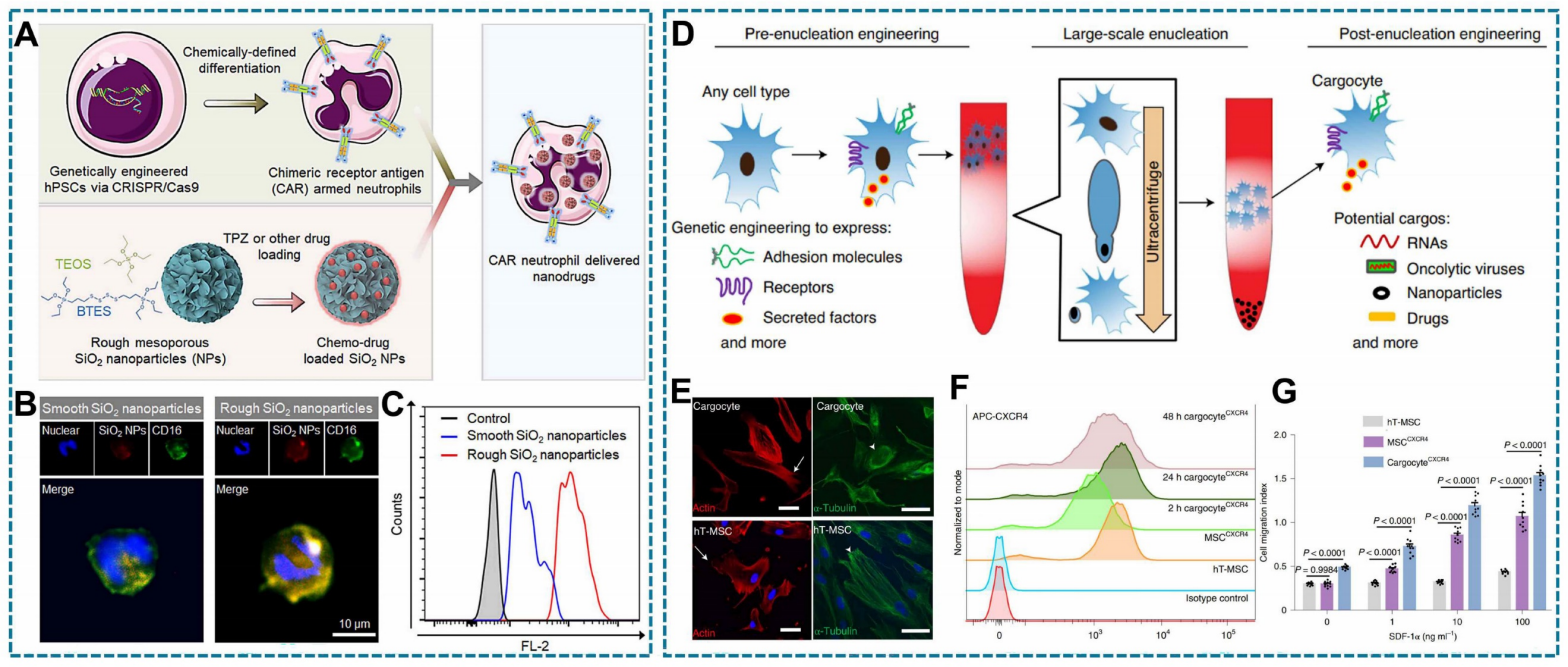
Relevant illustration of drug loading techniques involving genetically engineered.** (A)** Human pluripotent stem cells were engineered with CARs and differentiated into CAR-neutrophils that are loaded with rough silica nanoparticles containing hypoxia-targeting tirapazamine or other drugs, as a dual immunochemotherapy. **(B-C)** The neutrophils loaded with smooth and rough SiO_2_-TPZ NPs, and detected significant cellular uptake of rough SiO2-TPZ NPs than smooth SiO_2_-TPZ NPs via fluorescence microscope and flow cytometry analysis. (A-C Reproduced with permission 67 copyright 2023, Springer Nature) **(D)** Schematic of workflow for therapeutic uses of bioengineered enucleated cells (Cargocytes). **(E)** Fluorescent confocal images show that the hT-MSC-derived Cargocytes (“Cargocytes”) maintain well-organized cytoskeletal structure. The hT-MSCs/Cargocytes stained with rhodamine phalloidin for F-actin cytoskeleton (left), or anti-α-Tubulin antibody for microtubule network (right), and Hoechst for nucleus. **(F)** Graph shows cell surface expression of CXCR4 by flow cytometry. MSC ^CXCR4^, CXCR4 lentivirus-engineered hat-MSC; 2hr/24hr/48hr Cargocytes, MSC^CXCR4^-derived Cargocytes analyzed at indicated time points post-enucleation. **(G)** MSCs/Cargocytes migrated in Boyden chambers towards the indicated concentrations of SDF-1α for 2hr. Bar graph represents the cell migration index (migrated MSCs/Cargocytes versus loading control) (D-G Reproduced with permission 12 copyright 2023, Springer Nature).

The cell viability and function will be influenced under pathological microenvironment. Therefore, reprogramming cells to adapt to the pathological microenvironment through genetic engineering is also promising in the study of LCDDS. Chimeric antigen receptors (CAR) are a common technology in tumor therapy, most notably CAR-T cells. CAR genes are transduced into cells via gene vectors and are designed to recognize tumor antigens 189, 190. Genetically engineering the living cells with disease-specific CAR could redirect their functions against inflammation, and stimulate immune response 191. In addition, stem cells have multidirectional differentiation potential, which can be genetically engineered to differentiate into desired cells that express therapeutic biomolecules. The combination of CAR and stem cells makes stem cells-derived living cells to be a desirable solution to combat various diseases. For example, by chemical induction, a clustered regularly interspaced short palindromic repeats (CRISPR/Cas9) system-edited CAR-expressing hPSCs were differentiated into CAR-neutrophils for delivery therapeutic agent, in which the CAR-neutrophils could maintain an anti-tumor phenotype thus enhancing anti-glioblastoma activities. Because neutrophils preferentially phagocytose particles with rough surface, researchers designed a triapazamine (TPZ) loaded and rough surface organic silica (R-SiO_2_) nanoparticles to incubate such CAR-neutrophils, constructed a CAR-neutrophils DDS (Figure 7A) 67. And compared with smooth-SiO2-TPZ, the neutrophils show more significant cellular uptake of rough-SiO2-TPZ (Figure 7B-C) 67. Engineered cells are created by directly introducing the CAR gene into cells, allowing for stable expression of tumor-specific antigens on the cell surface. For example, researchers have used genetic engineering to make RAW264.7 macrophages stably express affinity protein targeting human epidermal growth factor receptor 2 (HER2), thereby producing engineered macrophages. This affinity protein specifically anchors to the cell membrane outside of the modified macrophages, allowing the engineered macrophages to recognize and bind to HER2-positive cancer cells 135. While, some studies suggest that living cells respond to external stimuli, which may cause unpredictable side effects. Therefore, to eliminate this effect, miRNA encapsulation in enucleated MSCs has been investigated for delivery (Figure 7D) 12. Importantly, the enucleated cells (Cargocytes) have complete cytoskeleton (Figure 7E). Furthermore, the functional CCR4 chemokine receptors of Cargocytes are up-regulated, indicating they have a stronger chemotactic ability (Figure 7F-H) 12.

As a personalized treatment, genetically engineered cells can formulate specifical cells and achieve exact treatment. However, the risk of gene editing technologies is a potential threat to their clinical transformation. On the one hand, gene-off and non-specificity may occur *in vivo* thus inducing serious side effects. On the other hand, genetically engineered cells have the features of genetic instability and low nucleic acid uptake, which further trigger mutation and uncontrolled genetic expression. Additionally, the selection of gene vectors is required to be non-toxic. Finally, gene technology is complex and challenging, hence cells with short lifespans such as neutrophils are limited to genetically engineered modifications.

To achieve more precise therapeutic effects, it is important for living cells co-delivery different drugs or genes for synergistic therapy. However, the differences in pharmacokinetics among various drugs may result in some changes in potency or competition for binding sites, thus leading to increased free drugs and affecting their efficacy. Furthermore, low loading efficiency induced by potential drug instability in the cellular environment or using inappropriate loading techniques that could lead to drug degradation needs to improve. Lastly, considerations regarding biocompatibility and toxicity are crucial as off-target effects and over-loaded drugs may trigger immune responses and toxicity.

### Controlled drug release of LCDDS

**Figure 8:**
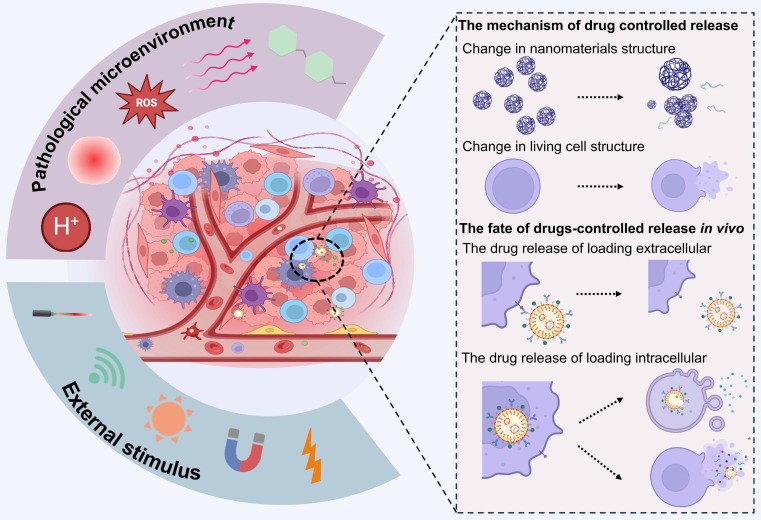
The mechanisms underlying drug controlled release and their *in vivo* fate (Figure was created with BioRender.com).

Following their entry into the human body, responsive nano drugs form nanoparticle-protein coronas that confer biological properties distinct from those of synthetic drugs, which renders them susceptible to capture and degradation. Moreover, drugs have a limited ability to penetrate biological barriers, which consequently confines them to the superficial areas of the disease site. While the primary goal of LCDDS is to achieve targeted drug delivery to the deeper regions of pathological lesions while overcoming the challenges posed by biological barriers and minimizing the off-target effects, which rely on the inherent physiological functions of the cells they are prepared with. Achieving an optimal drug release profile is the fundamental prerequisite for mitigating toxicity and enhancing therapeutic efficacy. This section primarily focuses on the mechanism of drug release and the *in vivo* fate of LCDDSs (Figure 8).

### The mechanism of drug controlled release

The LCDDS with responsive property is composed of living cells loading responsive materials 178, which can control drug release. These materials are sensitivity towards pathological microenvironment and external stimulation. Therefore, effectively harnessing the response signals of disease site could achieve precise release.

When drugs are located on the surface of living cells rather than inside them, the pathological microenvironment and exogenous stimulus generally trigger drugs' structural changes and lead to drug release. This phenomenon has led to extensive development of a variety of responsive materials. Pathological tissues have elevated levels of ROS and glutathione, but reduced pH levels, hypoxia, and inflammatory signaling. The typical hydrolysable or protonated moieties of pH-sensitive groups enable them to respond to changes in the environmental pH, which induces significant alterations in the concentrations of ions in the intracellular and extracellular compartments 192. The oxidation-reduction environment of diseased tissues can induce redox reactions in drugs or certain chemical moieties, which leads to structural modifications, rapid disassembly of the drug carrier, and release of the encapsulated drug 193, 194. In conclusion, stimulus-responsive drugs will change when expose to pathological microenvironment. It causing structural changes and release drugs through reactions such as bond breaking/formation, swelling, molecular dissociation, degradation, disruption of hydrophilic/hydrophobic balance, and other alterations 195. For example, drug loaded on the cell surface by host-guest chemistry, the guest ferrocene has an oxidation-responsive property, which will decompose the host-guest complex in oxidative conditions, and causing the modified drugs detach from the cell surface 160.

Exogenous stimuli such as light, near-infrared radiation, magnetic fields, or ultrasound can be employed not only in response to pathological factors at the lesion site but also for precise spatiotemporal control over drug release from the external environment 196. This responsive release method also alters the morphology and structure of drugs, and directly interacting with cells. And exogenous stimulation by light radiation and magnetic fields generates heat and releases cascade reaction signals, which convert light energy or magnetic energy into thermal energy or ROS, thereby indirectly destroying the material assembly 197, 198. The ultrasonic oscillation causes material structural changes via sonoporation 120. Additionally, narrow arterial blood vessels have significant shear stress 199, which may lead to drug detachment from cell surfaces targeting vascular stenosis diseases such as atherosclerosis 199 or tumor lung metastases 200.

In addition, under normal circumstances, the integrity of cellular membranes is crucial for intracellular homeostasis and protection against pathological microenvironments and external stimuli. However, cells may experience permeability and energy imbalance when expose in abnormal factors such as oxidative stress and light-heat stimulation 201. This strategy can also lead to imbalances in membrane permeability and energy in addition to promoting abnormal signal transduction, which may trigger cellular stress responses and ultimately lead to alterations in cellular structure 202. The drug loaded within carrier cells can therefore be released via the destruction of the cell membrane in response to pathological or exogenous stimuli.

**Figure 9:**
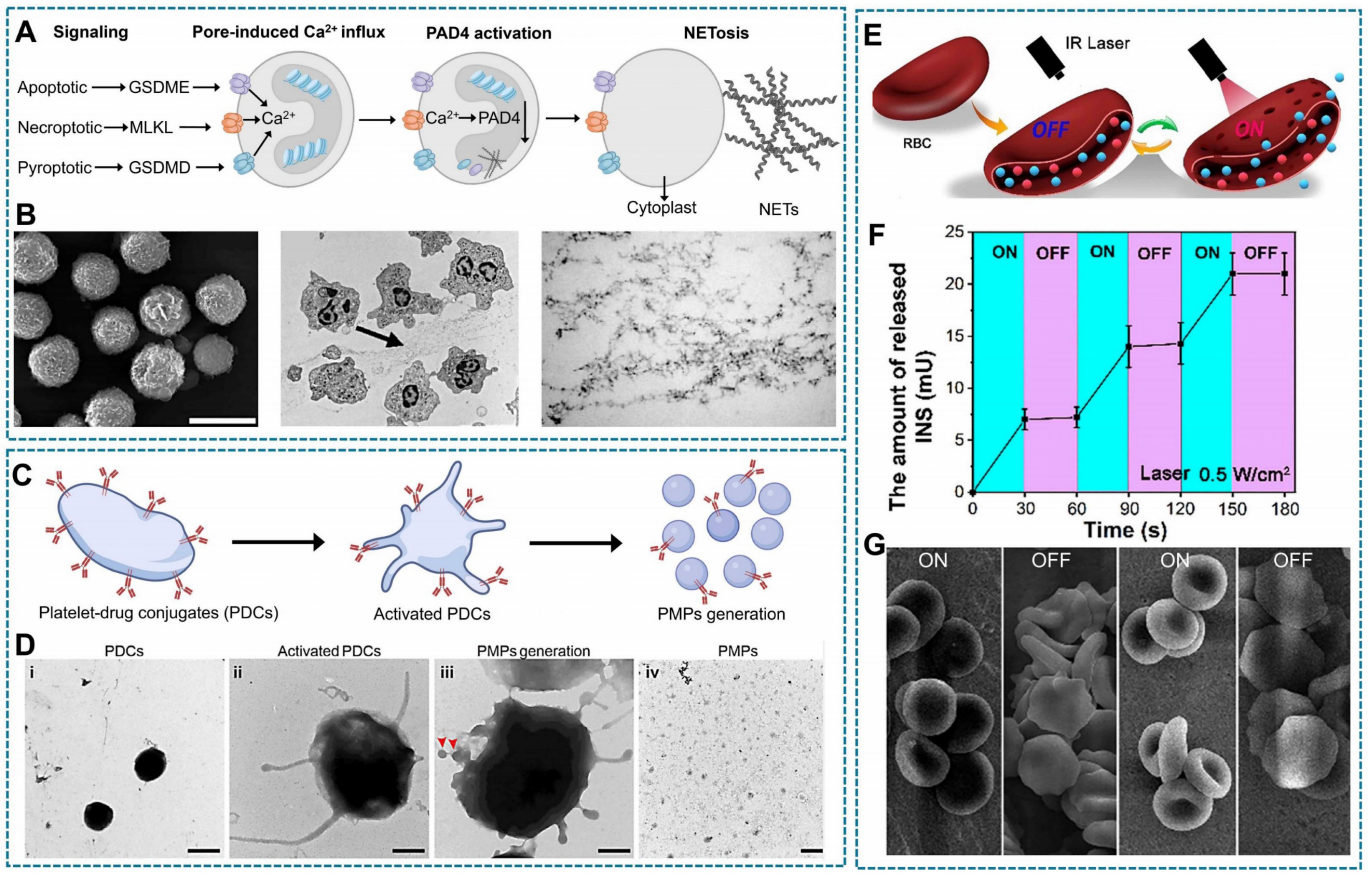
Relevant illustration of drug controlled release through NETs, PMPs, and damage to cell membrane structures.** (A)** The progression for a neutrophil from apoptosis to NETosis. When a healthy neutrophil receives apoptotic, necroptotic, or pyroptotic stimuli, the cell will open a pore in the membrane (GSDME, MLKL, or GSDMD, respectively). These pores permit calcium influx, causing PAD4 activation, which converts positively charged arginine residues in histone to neutrally charged citrulline. This causes DNA to unwind from histones. Given enough time, PAD4 activity causes the neutrophil to extrude its DNA in the process of NETosis, forming NETs. Created with BioRender.com. **(B)** Resting neutrophils are round and devoid of fibers (left), The activated cells in (middle) have many pseudopods and show NETs (arrow). Analysis of cross sections of the NETs by transmission electron microscopy (TEM) revealed they were not surrounded by membranes (right). (Reproduced with permission 203 copyright 2004, The American Association for the Advancement of Science)** (C)** Schematic indicate PMPs released from the PDCs to activated-PLTs. Created with BioRender.com. **(D)** TEM images of (i) platelet-drug conjugates, (ii) activated PDCs, (iii) PMP generation, and (iv) PMPs from PDCs. Red arrows indicate PMPs (above). Confocal fluorescence images of Cy3-labeled platelet-drug conjugates pre-and post-activation (Reproduced with permission 48 copyright 2023, Elsevier). **(E)** The formation of the hydrogel in which laser radiation triggers the release of insulin (INS)^207^.** (F)** The *in vitro* release profile of INS when the laser was cyclically varied between on and off for several repetitions. The concentration of INS increased as the laser was turned on, while no more INS was released from INS-ICG@RBC hydrogel when the laser was turned off 207.** (G)** The morphological changes of INS-ICG@ER during the “on-off” INS release (E-G Reproduced with permission 207 copyright 2023, Elsevier).

Neutrophils, within their limited lifespan, migrate from the bloodstream to infected tissues, where they efficiently bind, engulf, and inactivate bacteria 203. Besides, they secrete granules containing cytotoxic enzymes and form NETs composed of granular proteins, cytoplasmic components, and antimicrobial factors through a process known as NETosis 204. The signals associated with apoptosis, necroptosis, and pyroptosis have been documented to induce NETosis and facilitate plasma membrane permeabilization through the action of pore-forming molecules gasdermin E (GSDME), mixed lineage kinase domain-like protein (MLKL), GSDMD on the cell membrane respectively. These channels enable the influx of extracellular calcium ions for activating protein arginite delminase 4 (PAD4). Activated PAD4 could citrullinate the histone protein resulting the unwinding and extrusion of DNA from the intracellular (Figure 9A-B) 205. The mechanism of NETs formation supplies a splendid idea for the controlled release of neutrophils-loaded drugs.

Platelets are dynamic cells with a distinctive morphology, it will release significant quantities of PMPs composed of plasma membrane upon activation by inflammatory signals (Figure 9C-D) 206. For example, sonodynamic therapy stimulate the platelets-loaded sonosensitizers metal-organic framework to generate ROS, which lead morphological change of platelets and the release of drugs 120. While, the other example is that macrophages-based DDS containing oxaliplatin and the photosensitizer Zinc phthalocyanine was found to induce increased permeability and rupture of macrophage membranes following heat generated from photodynamic therapy, which trigger the acceleration release of therapeutics 75.

The excellent fluidity, deformability and ductility of the RBCs membrane enable them to traverse smaller capillaries than their own size during blood circulation and eventually reach the designated position. Thus, under mechanical stress, the morphology changes of RBCs facilitate sustained release drugs 28. Furthermore, excessive exogenous stimuli such as excessive ROS and H_2_O_2_ can disrupt the integrity of the cellular membrane, thereby inducing intracellular drug release as well 118, 207. For instance, an injectable hydrogel, inspired by RBCs and activated by laser, was developed (Figure 9E). Upon laser irradiation, indocyanine green (ICG) can generate ROS leading to the opening of the RBCs' phospholipid bilayer and triggering the release of insulin (INS). However, cessation of laser irradiation results in the disappearance of the aforementioned phenomenon (Figure 9F-G) 117.

### *In vivo* fate of LCDDS

The morphology and structure of drugs and living cells both change in response to stimuli, resulting in different release routes on the basis of LCDDS.

Drugs loaded onto the surface of carrier cells are immediately released following alterations in the morphology and structure of the cells or drugs themselves, thereby leading to the direct targeting of the disease. For instance, the drug may be directly bound to the cellular surface via physical or chemical bonds, and the linker is usually designed in such a manner that it undergoes activation and releases the drug under specific conditions, such as in the presence of certain enzymes or at specific pH and temperature. The breakdown or alteration of the linker leads to the release of the drug from the surface of the cell and ensures its targeted delivery to the lesion 208. Another mechanism of drug release involves altering the cellular structure or the integrity of the cell membrane, which induces the detachment and release of the drug adhering to the cellular surface to enable targeted drug delivery to the disease site. For instance, neutral granulocytes designed to carry hypoxia inducible factor-1α inhibitors can release NETs upon external stimulation and effectively deliver the drug to the TME 209.

The drugs that are encapsulated within cells can also be released intracellularly, thereby preserving the integrity of the cellular structure. On one hand, the drugs that are released intracellularly can modulate cellular functions to induce the secretion of EVs and cytokines that lead to the indirect remodeling of the microenvironment for treating diseases. For instance, nanomicelles containing the antioxidant resveratrol release resveratrol within mitochondria following phagocytosis by neurogliocyte to mitigate oxidative stress by eliminating the excess ROS. The released resveratrol also alleviates neuroinflammation by inducing the polarization of M1 macrophages towards the M2 phenotype 210. On the other hand, nanomedicines can also induce the establishment of micron-scale assemblies via host-guest binding 14 or biologically orthogonal functional groups 211, to mitigate the potential adverse effects resulting from drug leakage. For instance, phagocytes perform endocytosis in a step-wise manner following exposure to nanoparticles modified to carry cyclodextrin or amantadine. It has been reported that the formation of micrometer-scale assemblies within cells is facilitated by the strong host-guest binding between cyclodextrin and amantadine 14. It has been additionally demonstrated that these micrometer-scale drugs affect cellular function. The release of the drug and cellular contents is mediated by corresponding structural changes in cells in response to specific stimuli. Previous studies have aimed to design RBCs permeated with thrombin and TPZ via hypotonic or hypertonic treatments, and subsequently compromised the integrity of the RBC membrane by laser irradiation to release the contents 20. Rather than releasing the drug intracellularly and subsequently releasing the cellular contents, stimulus-responsive nanomedicines are released simultaneously during the release of cellular contents to maintain the efficacy of the drug.

The stimulus-response nanoplatforms exhibit significant potential in facilitating precise and controlled release of LCDDS at disease sites. However, the drug may be affected by other areas and lead to early release. Therefore, considering the synergistic effects of multiple stimulus responses can enhance targeting efficiency. It is crucial to achieve a balanced depth of exogenous stimulation to avoid shallow penetration of the drug into deep tissues. It is also necessary to avoid excessive stimulation that may result in cell death at non-disease sites. Although cells exhibit a certain degree of chemotaxis, the use of cells as drug carriers may limit the transport of the drug to the target site and result in uncontrolled off-target accumulation owing to their insufficient transport capacity 13. Additionally, cells have certain limitations as drug carriers that need to be overcome to ensure precise targeting. For instance, LCDDSs need to overcome shear stress caused by the blood flow during their transportation to the target organ, which in turn increases the transport time; however, cell-based micro motors can empower cells to achieve sustained self-propulsion, thus enabling active and mobile delivery 15. The attachment of specific antigen domains to cells can increase the chances of specific binding between cells and target tumors. For instance, transferring the CAR gene with CD133 antigen domain to macrophages, redirecting the phagocytosis function of macrophages towards tumors and stimulates adaptive anti-tumor immune responses 191. Additionally, downregulation of the expression of inflammatory factors is expected to diminish the efficacy of migration of live cells. Therefore, emerging strategies aimed at enhancing the targeting efficiency of carrier cells by modulating the microenvironment of the target tissue to amplify the homing signal of cells has gained traction. For instance, in photothermal therapy, tumor cells are induced to undergo apoptosis and release inflammatory factors, which not only stimulates the generation of more neutrophils from the bone marrow, but also augments their migration to the site of inflammation 130.

Nevertheless, evaluating the in vivo fate of LCDDS in animal models is one of the most substantial challenges, including drug potency, half-life and biodistribution, which is imperative for its development and clinical application. Intravenous injection is commonly employed for drug administration route in animal experiment due to its rapid systemic distribution. Various innovative methods have been developed to improve the targeting of LCDDS. For example, the antitumor bacterial toxin and arginine-glycine-aspartic acid peptide (RGD) are used to arm macrophage for fabricating a dual engineered macrophage-microbe encapsulation. Subsequently, the in vivo imaging confirms lung metastases targeted delivery due to chemotaxis and RGD-mediated adhesion 136. The other hand, metabolism is a crucial process for alleviating the potential risks. Endogenous cells hijack drugs following targeted delivery, and then the senescent carrier cells are eventually cleared. This process may generate recycled decomposition products, lead to the release of cytokines and cause tissue damage 212 Meanwhile, the surviving cells may rejoin the immune response. As for exogenous cells, apart from undergoing similar metabolic pathways, they may also be recognized and cleared by the immune system, potentially eliciting an immune response that can impact the metabolism and clearance of drug.

## Clinical trials of LCDDS

LCDDS have developed for decades with remarkable results. Currently, RBCs and stem cells are undergoing clinical trials to evaluate their potential therapeutic benefits (Table 3). The promising results highlight the efficacy of LCDDS as a targeted therapy strategy and its potential to expand the scope of clinical applications.

RBCs-based DDS, as the first cell carrier to enter clinical trials, has emerged as a promising treatment option for genetic defects, autoimmunity, and cancer by improving the pharmacokinetics of Dexamethasone-21-phosphate, L-Asparaginase, Phenylalanine ammonia lyase, and Thymidine phosphorylase. In 2003, a Phase II clinical trial demonstrated that the efficacy and safety of RBCs mediated delivery of dexamethasone 21-phosphate in patients with steroid-dependent ulcerative colitis without steroid-related adverse effects (NCT01171807) 213. Subsequently, phase III trial was conducted in 2009 to evaluate the long-term therapeutic potential of dexamethasone in RBCs, but result proved unable to prevent disease recurrence after stopping drugs and was discontinued due to difficulties in enrolling suitable patients (NCT01277289) 213. In 2011, RBCs loaded dexamethasone 21-phosphate was used to evaluate the effect of neurological symptoms with ataxia telangiectasia in phase II (NCT01255358). And the object of phase III trial is to gather information on the long-term safety and effectiveness of treatment ataxia telangiectasia in 2018, but there was no follow-up (NCT03563053). The efficacy of RBCs-mediated dexamethasone 21-phosphate in reducing inflammation following coronary stenting remains uncertain in a phase IV clinical trial (NCT00484965).

In acute lymphoblastic leukaemia (ALL), phase I/II trials compare the administration of RBC-load L-asparaginase with natural L-asparaginase, which was aimed to investigate the relationship between RBC-load L-asparaginase and duration of asparagine depletion (NCT00723346). The main purpose of Phase II study is to determine the maximum tolerated and efficient dose of RBCs encapsulating L-asparaginase (NCT01523782) and Phase II/III study evaluating efficacy and safety in patient with ALL (NCT01518517). Besides, asparaginase in RBCs is also used in patients with PEG-asparaginase allergy (NCT03267030, phase II) and lymphoblastoma (NCT01910428, phase I) and pancreatic adenocarcinoma (NCT01523808, phase I and NCT02195180, phase II). A new phase II clinical trial is being conducted to determine the safety, tolerability and efficacy of RBCs-coated thymidine phosphorylase in patients with mitochondrial neurogastrointestinal encephalomyopathy (NCT03866954) 214, but the research progress was unknown because of insufficient patient populations. With the rapid genetic engineering, Red-cell therapeutics (RCT) began to enter people's field of vision. *Rubius* uses HSCs and gene editing technology to obtain mature RBCs. In 2020-2023, RBCs (RTX-224, RTX-321, RTX-240) were first used to express 4-1BBL and IL-12/ IL-15, and then CD8^+^ T cells and NK cells were stimulated to treat solid tumors (NCT05219578), acute myeloid leukemia (NCT04372706) and HPV-16 positive tumors (NCT04672980) 102, but the trial was terminated due to difficulties in recruiting patients and safety concerns.

Stem cells is crucial in the advancement and infiltration of neoplastic growths. In recent years, a series of clinical trials on the basis of stem cells DDS have been conducted, which mainly focus on carrying prodrug invertase 215, apoptosis-inducing factor 216, and oncolytic viruses 217, 218 to migrate to the tumor site through cell engineering technology. Besides, NSCs and MSCs are commonly used to deliver anti-cancer drugs to tumors. These trials have been completed that both genetically-modified NSCs load prodrug invertase (NCT01172964, phase I) and oncolytic adenovirus (NCT03072134, phase II). The NSC loaded with prodrug invertase to determine preliminarily the safety and feasibility. And NSC loaded with the oncolytic adenovirus to assess the response of tumor treatment and determine the maximum tolerated dose for a future phase I study. In a phase I/II trial (NCT02008539), MSCs genetically modified to express the carboxylesterase were safety and tolerability in advanced gastrointestinal cancer. In a recent report, expressing tumor necrosis factor-related apoptosis-inducing ligand (NCT03298763, phase I/II), interferon-β (NCT02530047, phase I), Thyroidal sodium iodide symporter-expressing oncolytic measles virus (NCT02068794, phase I/II), *etc.* It derived from MSCs engineered and facilitate the effective killing tumors. In addition, Fabry disease is a genetic disease caused by alpha-genetic mutation of alpha-galactosidase A (alpha-Gal A), and a phase I (NCT02800070) trial transferred the alpha-Gal A gene lentiviral vector into stem cells. It wants to confirm the safety and efficacy of stem cell transplantation with alpha-Gal A gen, but it is no obvious research progress for some reasons 219.

Since RBCs and stem cells enter into clinical trials, indicating their potential effect on DDS. The RBCs has matured intracellular encapsulation techniques, and emerging surface coupling and genetical engineering techniques in clinical trials. Significantly, modifying drugs on RBCs' surface may interfere with the signal of CD47/SIRPα axis, it will result in RBCs malformation and reduce the osmotic fragility of RBCs. And RCT therapy induce immature RBCs to express therapeutic proteins, as well as shows promising safety and tolerability results in phase I/II clinical trials. But the efficacy is marginally unacceptable, immature technology and difficult finance result in RCT failure. The technology of stem cells-based DDS application in clinical trials is mainly cell engineering for tumors treatment, including viral-mediated transduction and gene editing technologies 101. But these clinical trials are still in the early stages. It is critical to reduce immunogenicity of stem cells-genetical modified to provide long-term therapeutic effects and extend new therapeutic frontiers. Meanwhile, the study of macrophages and neutrophils are not enough and has not yet reached the clinical stage. Importantly, Platelets-based DDS is about to clinical trials under the joint efforts of *Gu's* team 220. Although these clinical trials have experienced varying degrees of success and failure, the main failed reasons are inadequate safety, insufficient patient recruitment and limited technology. In the future, it is not only necessary to expand and improve the existing cell carriers for the treatment of various clinical diseases, but also to develop more optimized and safe technologies. We believed that the emergence of advanced technologies and applications will accelerate the speed of cell drug delivery therapies into the clinic.

## Conclusion and Outlook

**Figure 10:**
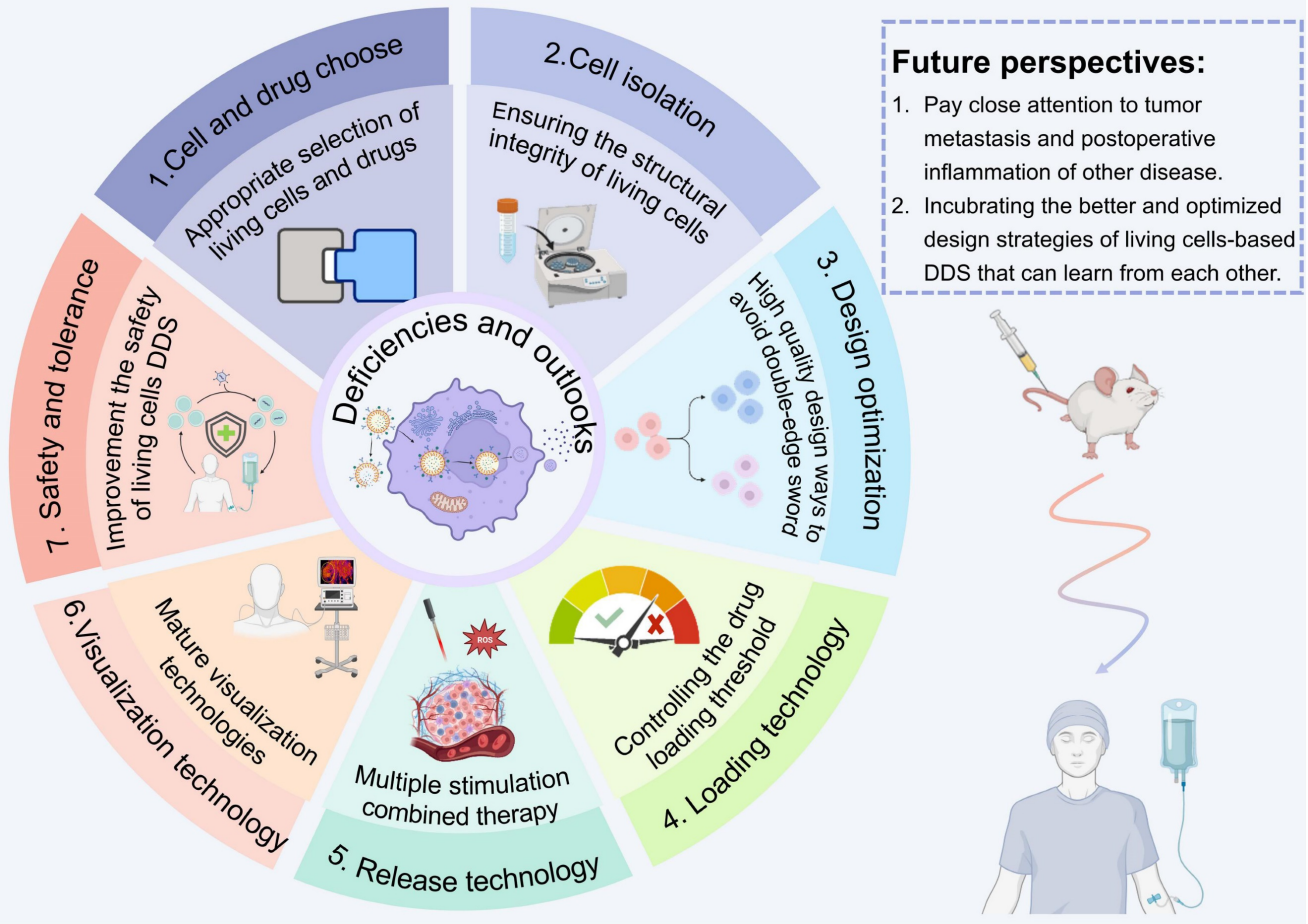
Conclusion and perspective of LCDDS (Figure was created with BioRender.com).

Compared to traditional DDS, numerous studies have demonstrated that LCDDSs have a greater potential for application in drug delivery owing to their unique biological properties and functions. The distinctive biological properties of living cells enable efficient drug loading. For instance, stem cells with multidirectional differentiation potential can be genetically engineered for modification. Neutrophils and macrophages possess natural phagocytic activities which enable them to engulf drugs, while RBCs can be loaded with drugs by exposing them to hypotonic treatments that cause osmotic fragility. Additionally, living cells can actively target sites of inflammation and exhibit immunomodulatory properties, especially in pathological areas that are hard to reach, thereby aiding in the therapeutic management of the disease. These findings demonstrate that the utilization of living cells as drug carriers would enable the application of the enhanced functions of living cells and augment the therapeutic effects of drugs, thus resulting in a synergistic therapeutic effect. The development of LCDDSs is still at an early stage at present, and despite significant improvements in therapeutic efficacy, uncertainties regarding their safety and feasibility under unknown *in vivo* microenvironments hinder their clinical translation. Considering the challenges in the current developmental status of LCDDSs, it is proposed that future research should focus on enhancing their therapeutic efficacy (Figure. 10).

1) Cell and drug choose: The various types of living cells present in the complex *in vivo* environment of disease sites can actively sense the microenvironment and participate in immune responses. It is therefore necessary to consider the intrinsic functions of these different cell types and select appropriate drugs for the therapeutic management of diseases. The presence of inflammatory cytokines and ROS in the disease microenvironment can decrease cellular viability and potentially hinder the targeting and therapeutic efficacy of DDSs. The development of a versatile and modular live cell-based DDS is therefore crucial to ensure that the loaded drugs effectively eliminate local inflammation while preserving cellular viability. It is important to note that living cells may induce the immune response, thus functioning as a double-edge sword. This indicates that high-quality control schemes are necessary for optimizing the advantages of living cells and compensating for their shortcomings.

2) Cell isolation: Previous studies have primarily aimed to construct cell-based drug carriers *in vitro* prior to their *in vivo* administration. This necessitates the isolation and amplification of cells, implying that the cellular integrity could be destroyed during the process. Additionally, the cells that have a short life span need to be rapidly isolated following separation and preparation to prevent a reduction in activity, indicating that the technology available for these cells is limited.

3) LCDDS design strategy: Platelets, neutrophils, macrophages, and stem cells possess a dual nature in that they can exhibit both pro-inflammatory and anti-inflammatory phenotypes under specific conditions. It is therefore imperative to implement high-quality control measures for optimizing their advantages and compensating for their shortcomings. For instance, the anti-tumor phenotype of neutrophils can be enhanced via the identification of cytokines or the application of genetic engineering techniques. As living cells exhibit intricate interactions, the development of various living cells-based DDS can potentially enhance the widespread applicability of cell-drug conjugates in the treatment of various diseases. Researchers should therefore focus on other pathological changes beyond primary lesions, including disease complications, tumor metastasis and recurrence, and postoperative inflammation, and strive to develop novel cell-based drug delivery platforms to alleviate these conditions.

4) Drug loading technologies: Drugs loaded extracellularly on the cellular surface may interfere with the cellular signal transduction pathways, affect intercellular communication, and may be cleared by phagocytes. Furthermore, these drugs may cause off-target side effects and exhibit non-specific binding to non-target cells. Additionally, nanomedicines loaded on the surfaces of cells may form nanoparticle-protein coronas that affect cellular activity. Although intracellularly loaded drugs can overcome the issues faced by extracellularly loaded drugs, they have the disadvantage of undergoing degradation in the intracellular compartment. As surface coupling is difficult for phagocytes, it is necessary to identify the balance between phagocytosis and anchoring technologies by selecting the optimal reactions and lipid materials. Finally, the threshold of drug loading should be considered for cell-based DDSs, as drugs loaded below the threshold are ineffective, and drug loading above the threshold affects the vitality and physiological functions of cells. Therefore, the combination of extracellular and intracellular loading provides an optimum strategy for drugs that necessitate administration at high doses. It is also necessary to develop novel loading technologies that do not affect cell viability or the stability of the loaded drugs.

5) Drug controlled release techniques: it is necessary to identify novel strategies to prevent the untrolled release of cell-drug conjugates at present. The challenge can be solved by combing with drug conjugation methods.

The controlled release of drugs can also be achieved by generating local hyperthermia or ROS using external stimulation, failing which the uncontrolled release of drugs is likely to destroy other tissues. The application of multiple stimulus-responsive materials for the precise treatment of diseases is worthy of attention in future research.

6) Drug visualization techniques: Although LCDDS has shown remarkable progress and periodic success in some animal models, there are still differences in human. And there are relatively few studies devoted to the* in vivo* fate of LCDDSs. Hence, it is critical to establish more mature visualization techniques to demonstrate the final drug delivery efficiency.

7) Safety of LCDDS: Although LCDDS is regarded as personalized therapy, and cellular sources have gradually evolved from autologous to allogeneic, the safety and tolerance of LCDDSs require further attention. The number of immune cells is likely to decrease* in vivo* following their isolation, which can potentially suppress the immunity of the host in general. In addition, cells loaded with nanomedicines are potentially toxic. On the other hand, although genetically engineered cells are endowed with additional functions, it is necessary to elucidate the safety of genetic engineering techniques for precisely inducing the differentiation of stem cells. Furthermore, the processes involved in the production of LCDDS comprise several intricate steps that involve separate standardization measures for enhancing the clinical translatability of LCDDSs.

Altogether, the review highlights that the advantages of LCDDSs in terms of application and safety cannot be ignored, despite several challenges. It is believed that LCDDSs will serve as effective platforms in future and provide therapeutic solutions for diseases while enabling the investigation of unexplored domains.

## Figures and Tables

**Table 1:** Comparisons the characteristics of various drug delivery platforms

Platforms	Advantages	Disadvantages
NDDS	Improve the solubility and stability of the drugs; Nanocarriers achieve muti-functional design; Initially achieve effective drug loading, active/passive targeting and responsive drug release.	Potentially immunogenicity as an exogenous substance; The formation of protein corona will occlude NPs' surface properties and affect the behavior, which reduce the targeting and rapid immune clearance; Low manipulation in vivo and lack of intelligence (active perception of pathological environment in vivo).
EBDDS	Reverse the surface composition, shape, and movement of normal cellular physiology; Immune escape, long blood circulation time, specific molecular recognition and cell targeting.	Low productivity; Incomplete cell membrane and leads to cargo leakage.
LCDDS	Excellent biocompatibility, long circulation, low immunogenicity and individual-based treatment; Various drug delivery methods; Actively sensing the complex microenvironment in vivo and crossing biological barriers; Synergistically treat diseases with the loaded drugs.	High cost of cell engineering; Drug loading, drug release technology, and cell fate in vivo monitoring technology need to be further studied.

Note: NDDS, nano-drug delivery systems; EBDDS, endogenous biomaterials drug delivery system; LCDDS living cell drug delivery systems.

**Table 2:** Summary the characteristics of different cells and their application mechanism

Cells	Prolong half-life	Physiological functions	Regenerative functions	Anti-inflammation	Pro-inflammation	Inflammation homing	Bone marrow homing	Others	Ref.
RBCs	✔	✔		✔			✔	Large volume space and long circulation time; Transport of nutrients and oxygen.	21, 26, 31
Platelets	✔	✔		✔	✔	✔		Formation of platelet microparticles.	38, 47, 52
Neutrophils		✔		✔	✔	✔	✔	Abundant in quantity and rapid response to inflammation; Having two phenotypes: N1 (anti-tumor) and N2 (pro-tumor); Formation of NETs.	57, 60, 109
Macrophages		✔		✔	✔	✔		Having two phenotypes: M1 (pro-inflammation and anti-tumor) and M2 (anti-inflammation and pro-tumor).	110, 111
Stem cells		✔	✔	✔		✔		Low immunogenicity and immunoregulatory properties.	86, 98, 112

**Table 3:** Examples of cell-based drug delivery carriers organized by cell type

Cell	Drug loading technology	Drug/Nano-pharmaceuticals	Controlled release	Application	Ref.
RBCs	Hypotonicity	Paclitaxel	pH-responsive	lung carcinoma Osmotic fragility	113
	Hypotonicity	Thrombin; Tirapazamine	Light-responsive	breast cancer	20
	Hypotonicity	Berberine hydrochloride	Sustained release	Bacillary dysentery	114
	Hypotonicity	Filamentous hemagglutinin	Magnetic-responsive	Lung Inflammation	115
	Electrostatic interaction	Vitamin K1	N/A	Long-acting anticoagulant rodenticides	116
	Adsorption	Nanoparticles	Change in cell structure	Pulmonary embolism	27
	Extrusion/sonication, phagocytosis	Dimercaptosuccinic acid	N/A	Chromium poisoning	31
	Adsorption	7-ethyl-10-hydroxycamptothecin	N/A	Colorectal adenocarcinoma	23
	Covalent binding	Mg sortase A and Pro-urokinase-type plasminogen activator	N/A	Thrombotic disorders	28
	Hypotonicity	Exogenous insulin	Light-responsive	Diabetes	117
	Hypotonicity	Endostar	Glucose-responsive	Breast cancer	118
	Carrier-Mediated Transport	Insulin; Glucose oxidase; Catalase	Change in cell structure	Type 1 diabetic	119
Platelets	Electrostatic interaction	α-methyl-DL-tryptophan	US/GSH-responsive	Breast cancer	120
	Ultrasound,	Interleukin-10	Inflammatory signaling activation	Unilateral ureteral obstruction; Ischemia/reperfusion injury	54
	N/A	Dimethyl fumarate	N/A	Multiple sclerosis	51
	Ligand-receptor	Tanshinone IIA	Matrix metalloproteinases 2-responsive	Murine pancreatic cancer	121
	Endocytosis	Doxorubicin	pH-responsive	Lymphoma	122
	Endocytosis	Chlorine e6; Doxorubicin	Light-responsive	Glioblastoma	123
	Lipid insertion; Endocytosis	Urokinase; Arginine	Inflammatory signaling activation	Carotid arterial thrombosis	124
	Covalent conjugation	anti-programmed cell death-ligand 1	Inflammatory signaling activation	Breast metastatic tumor; Melanoma	44
	Electrostatic interaction/Hydrophobic interaction	Perforin and granzyme B	Inflammatory signaling activation	Breast metastatic tumor	48
	Covalent conjugation	Anti-programmed cell death-ligand 1	Inflammatory signaling activation	Melanoma	45
	Genetic engineering	Anti-programmed cell death-ligand 1	Platelets activated by thrombin	Non-obese diabetic	38
	Covalent conjugation	Anti-programmed cell death-ligand 1	Inflammatory signaling activation	Acute myeloid leukemia	125
Neutrophils	Ligand-receptor	Ligustrazine.	ROS-responsive	Cerebral ischemia-reperfusion injury	60
	Receptor-mediated endocytosis	Glucose oxidase	N/A	Endometriosis	126
	Phagocytosis	N/A	Cytolysis	Atherosclerosis	63
	Phagocytosis	Abraxane	Cytolysis	Gastric cancer	127
	Phagocytosis	Paclitaxel	Cytolysis	Malignant glioma	62
	Phagocytosis	Cabazitaxel; Teriparatide	Sustained release	Osseous metastasis tumor; Osteoporosis	65
	Phagocytosis	Carboxylate silver; Urokinase	Cytolysis	Thrombus	15
	Phagocytosis	Oncolytic bacteria	Cytolysis	Melanoma lung metastasis	128
	CAR-engineering	Tirapazamine; Temozolomide	GSH-responsive; Cytolysis	Glioblastoma	67
	Receptor-mediated endocytosis	Doxorubicin; Paclitaxel	Light-responsive	Lung carcinoma	129
	Phagocytosis	Nano-pathogens	Light-responsive; Cytolysis	Breast cancer; Colon adenocarcinoma	130
	Phagocytosis	Biomimetic nanase	Cytolysis	Ischemic Stroke	131
	Receptor-mediated endocytosis	Edaravone	N/A	Cerebral ischemia	61
Macrophages	Biotin-avidin interaction	Doxorubicin	N/A	Triple-negative breast cancer	132
	Endocytosis	Doxorubicin	Macrophage polarization	Triple-negative breast cancer	133
	Phagocytosis	Apoptotic bodies; Toll like receptor 9 ligand	Light-responsive	Lymphoma	76
	Phagocytosis	Doxorubicin	Sustained release	Lung metastasis of breast cancer	134
	Host-guest interaction	Quercetin	ROS-responsive	Acute pneumonia	71
	CAR-engineering; Phagocytosis	human epidermal growth factor receptor 2; Doxorubicin	N/A	Breast cancer;	135
	Phagocytosis	*Salmonella Typhimurium*	N/A	Hepatoma	17
	Transfection; Phagocytosis	*Salmonella Typhimurium* VNP20009; arginine-glycine-aspartic acid peptide	Macrophage polarization	Breast Cancer Lung Metastasis	136
	Transfection	Glial cell line-derived neurotrophic factor	Extracellular vesicles	Parkinson's disease	70
	Cellular Backpacks	Bacteria	Extracellular vesicles; Macrophage polarization	Breast cancer	137
	Phagocytosis	Oxaliplatin	Light-responsive; Macrophage polarization	Breast cancer	75
	Host-guest interaction	Curcumin	ROS-responsive; Macrophage polarization	Acute pneumonia	13
	Receptor-mediated endocytosis	Resiquimod	Protease-responsive	Lung metastasis of breast cancer	83
Stem cell	Adsorption	Doxorubicin	Light-responsive	Triple negative breast cancer,	138
	Metabolic glycoengineered	Luteolin	ROS-responsive	Ischemic stroke	99
	Covalent conjugation	Nucleoside analogues	GSH-responsive	Glioblastoma	106
	Transduction	Interleukin-2	Cytokine secretion	Skin cutaneous melanoma	95
	Adsorption	Fibroblast growth factor 19	Sustained release	Schemic hindlimb	139
	Phagocytosis	N/A	Light-responsive	Osteosarcoma	94
	Transfection	pDNA	Cytokine secretion	Prostate cancer	140
	Transfection	mRNA	Cytokine secretion	Acute pancreatitis; Acute inflammation	12

**Table 4:** Clinical trials based on LCDDS

Cells	Cargo	First post	Application	NCT number	Clinical status	Phase
RBCs	Dexamethasone 21-phosphate	2003	Inflammatory bowel disease	NCT01171807	Unknown	Phase II
	Dexamethasone 21-phosphate	2009	Inflammatory bowel disease	NCT01277289	Terminated	Phase III
	Dexamethasone 21-phosphate	2007	Prevention of Stent Restenosis	NCT00484965	Unknown	Phase IV
	Dexamethasone 21-phosphate	2011	Ataxia telangiectasia	NCT01255358	Completed	Phase II
	Dexamethasone 21-phosphate	2018	Ataxia telangiectasia	NCT03563053	Unknown	Phase III
	L-Asparaginase	2006	Acute lymphoblastic leukemia	NCT00723346	Completed	Phase I/II
	L-Asparaginase	2009	Acute lymphoblastic leukemia	NCT01523782	Completed	Phase II
	L-Asparaginase	2009	Acute lymphoblastic leukemia	NCT01518517	Completed	Phase II/III
	L-Asparaginase	2013	Acute lymphoblastic leukemia	NCT01910428	Terminated	Phase I
	L-Asparaginase	2017	Acute lymphoblastic leukemia	NCT03267030	Completed	Phase II
	L-Asparaginase	2009	Pancreatic adenocarcinoma	NCT01523808	Completed	Phase I
	L-Asparaginase	2014	Pancreatic adenocarcinoma	NCT02195180	Completed	Phase II
	L-Asparaginase	2013	Acute myeloid leukemia	NCT01810705	Completed	Phase II
	Phenylalanine ammonia lyase	2020	Phenylketonuria	NCT04110496	Terminated	Phase I
	RTX-240	2020	Solid tumors or Acute Myeloid Leukemia	NCT04372706	Terminated	Phase I/ II
	RTX-321	2021	HPV 16+ Tumors	NCT04672980	Terminated	Phase I
	RTX-224	2022	Solid tumors	NCT05219578	Terminated	Phase I/II
	Thymidine phosphorylase	2024	Mitochondrial neurogastrointestinal encephalomyopathy	NCT03866954	Unknown	Phase II
Stem cells	Cytosine deaminase enzyme	2010	High-grade glioma	NCT01172964	Completed	Phase I
	HSV-TK;	2013	Advanced gastrointestinal adenocarcinoma	NCT02008539	Terminated	Phase I/II
	Carboxylesterase	2014	High-grade glioma	NCT02008539	Not recruiting	Phase I
	Thyroidal sodium iodide symporter-expressing oncolytic measles virus	2014	Recurrent ovarian cancer/ Primary peritoneal cancer/ Fallopian tube cancer	NCT02068794	Recruiting	Phase I/II
	GX-051	2014	Head and neck cancer	NCT02079324	Unknown	Phase I
	Interferon-β	2016	Ovarian cancer	NCT02530047	Completed	Phase I
	Alpha-Galactosidase A	2016	Fabry Disease	NCT02800070	Not recruiting	Phase I
	Oncolytic virus	2017	Malignant gliomas	NCT03072134	Completed	Phase I
	TRAIL	2019	Lung cancer	NCT03298763	Recruiting	Phase I/II

Note: Information obtained from the website https://clinicaltrials.gov/

## Abbreviations

alpha-Gal A: alpha-galactosidase A
aPDL1: anti-programmed cell death-ligand 1
α-granules: alpha granules
APC: antigen-presenting cells
ATP: adenosine triphosphate
ALL: acute lymphoblastic leukaemia
BPs: backpacks
BEP: bulk electroporation
β-CD: β-cyclo- extrin
CD: cluster of differentiation
cGVHD: chronic graft versus host disease
CME: clathrin-mediated endocytosis
CTCs: circulating tumor cells
CuAAC: copper-catalysed azide-alkyne cycloaddition
CXCR4: C-X-C chemokine receptor 4
CFLFLF: cinnamyl-F-(D) L-F-(D) L-F
CLIC/GEEC: clathrin-independent/dynamin-independent endocytosis
CAR: chimeric antigen receptors
CRISPR/Cas9: clustered regularly interspaced short palindromic repeats
*C_m_*: capacitance of the cellular membrane
DDSs: drug delivery systems
DSPE: 1,2-Distearoyl-sn-glycero-3-phosphorylethanola mine
DBCO: dibenzyl cyclooctyne
DOX: doxorubicin
EBDDSs: endogenous biomaterials drug delivery systems
EVs: extracellular vesicles
Fc: ferrocene
FPR: formyl peptide receptor
FR: folate receptor
FEME: fast endophilin-mediated endocytosis
G-CSF: granulocyte colony-stimulating factor
GSDME: gasdermin E
HA-Mal: hyaluronic acid-maleimide
HER2: human epidermal growth factor receptor 2
HSCs: hematopoietic stem cells
hESCs: human embryonic stem cells
hiPSCs: human induced pluripotent stem cells
ICD: immunogenic cell death
IL-1α: interleukin-1α
ICG: indocyanine gree
INS: insulin
LBL: layer-by-layer
LPS: lipopolysaccharides
LCDDSs: living cells drug delivery systems
Lip: liposome
MA-STA: macrophage-streptavidin
MHC: major histocompatibility
MLKL: mixed lineage kinase domain-like protein
MMP-2: matrix metalloproteinases 2
MPS: mononuclear phagocyte system
MSCs: mesenchymal stem cells
MEP: microscale electroporation
NEP: nanoscale electroporation
NSCs: neural stem cells
NDDSs: nano-drug delivery systems
NETs: neutrophil extracellular traps
NP: nanoparticle
NPNs: nano-pathogens
OMVs: outer membrane vesicle
PAMPs: pathogen-associated molecular patterns
PD-L1: programmed death-ligand 1
PD-1: programmed death-1
PDCs: platelet-drug conjugates
PMPs: platelet microparticles
PSGL-1: p-selectin glycoprotein ligand-1
PSCs: pluripotent stem cells
PLGA: poly (lactic-co-glycolic acid)
PEG: polyethylene glycol
PAD4: pmpsrotein arginite delminase 4
RBCs: red blood cells
ROS: reactive oxygen species
*R_e_*: extracellular resistance
Ri: intracellular resistance
R-SiO2: rough surface organic silica
RCT: red-cell therapeutics
RGD: arginine-glycine-aspartic acid peptide
SIRPα: signal regulatory proteins alpha
STING: stimulator of interferon genes
SEM: scanning electron microscope
STA: streptavidin
TANs: tumor-associated neutrophils
TAMs: tumor-associated macrophages
TCEP: tris (2-carboxyethyl) phosphine
TK: thioketal
TMP: tetramethylpyrazine
TNF: tumor necrosis factor
TLR9: toll-like receptors 9
TME: tumor microenvironment
TSCs: totipotent stem cells
TPZ: triapazamine
